# Flux balance analysis predicts essential genes in clear cell renal cell carcinoma metabolism

**DOI:** 10.1038/srep10738

**Published:** 2015-06-04

**Authors:** Francesco Gatto, Heike Miess, Almut Schulze, Jens Nielsen

**Affiliations:** 1 Department of Biology and Biological Engineering, Chalmers University of Technology, Göteborg 41296, Sweden; 2 Gene Expression Analysis Laboratory, Cancer Research UK London Research Institute, London WC2A 3LY, United Kingdom; 3 Theodor-Boveri-Institute, Biocenter, Am Hubland, 97074 Würzburg, Germany; 4 Comprehensive Cancer Center Mainfranken, Josef-Schneider-Str.6, 97080 Würzburg, Germany

## Abstract

Flux balance analysis is the only modelling approach that is capable of producing genome-wide predictions of gene essentiality that may aid to unveil metabolic liabilities in cancer. Nevertheless, a systemic validation of gene essentiality predictions by flux balance analysis is currently missing. Here, we critically evaluated the accuracy of flux balance analysis in two cancer types, clear cell renal cell carcinoma (ccRCC) and prostate adenocarcinoma, by comparison with large-scale experiments of gene essentiality *in vitro*. We found that in ccRCC, but not in prostate adenocarcinoma, flux balance analysis could predict essential metabolic genes beyond random expectation. Five of the identified metabolic genes, *AGPAT6, GALT*, *GCLC*, *GSS*, and *RRM2B*, were predicted to be dispensable in normal cell metabolism. Hence, targeting these genes may selectively prevent ccRCC growth. Based on our analysis, we discuss the benefits and limitations of flux balance analysis for gene essentiality predictions in cancer metabolism, and its use for exposing metabolic liabilities in ccRCC, whose emergent metabolic network enforces outstanding anabolic requirements for cellular proliferation.

The regulation of metabolism has been recognised to be of central importance in cancer^1^,^2^,^3^. Several studies have collectively suggested that cancer selects for cell clones that have reprogrammed their metabolism, resulting in distinct cancer type-dependent metabolic phenotypes^4^,^5^,^6^,^7^,^8^,^9^,^10^,^11^. These programs enforce cancer cell dependence on specific flux distributions, and disruption of the underlying pathways mostly results in cell death^12^,^13^,^14^,^15^,^16^,^17^.

Under these premises, metabolic modelling using flux balance analysis (FBA)^18^ is the only approach that can predict the effect of genetic and environmental perturbations in the disruption of such metabolic phenotypes at the genome scale^19^,^20^, and applications of these models for studying cancer or metabolic diseases have been advocated^21^,^22^,^23^,^24^. Contrary to other systems biology approaches, FBA typically involves only limited fundamental assumptions (e.g., mass and charge balance in all reactions, and thermodynamically constrained reaction directionality) and little to no parameter fine-tuning (e.g., non-growth and growth-associated ATP maintenance), yet still allows for meaningful genome-wide predictions of gene essentiality in a variety of model organisms^25^,^26^, provided that a genome-scale metabolic model for the organism is available. Nevertheless, a number of algorithms are now available to infer the active metabolic network in human cells^27^,^28^,^29^,^30^,^31^,^32^,^33^,^34^, and the constraints required to formulate a plausible FBA can now be more readily obtained due the increased availability of high-throughput data. Despite these promising conditions, use of FBA to predict gene essentiality in cancer metabolism is still at its infancy, and besides the extensive theoretical formulations reported in the literature, few practical studies have so far benefited from the systematic analyses enabled by FBA-based studies^35^,^36^,^37^,^38^,^39^.

In this study, we critically and systematically assessed the benefits and limitations of FBA for performing genome-scale predictions of gene essentiality in cancer metabolism. In particular, we were interested in the use of FBA to expose metabolic liabilities in clear cell renal cell carcinoma (ccRCC), the most common form of kidney cancer^40^. This cancer type was chosen as because we have recently uncovered that it features a compromised metabolic network^41^. We also verified whether the accuracy of FBA extends to a second cancer type, prostate adenocarcinoma (PC) and analysed the essentiality of selected genes in metabolic models of non-malignant tissues. Our findings suggest that FBA is suitable to uncover essential genes in cancers whose emergent metabolic network enforces outstanding anabolic requirements for cellular proliferation. Hereby we demonstrate that ccRCC depends on the expression of *AGPAT6, GALT*, *GCLC, GSS*, and *RRM2B*, which, although essential for cancer cells, are potentially nonessential in normal cells.

## Results

### Strategy used to benchmark predictions of gene essentiality in cancer metabolism

**Figure 1:**
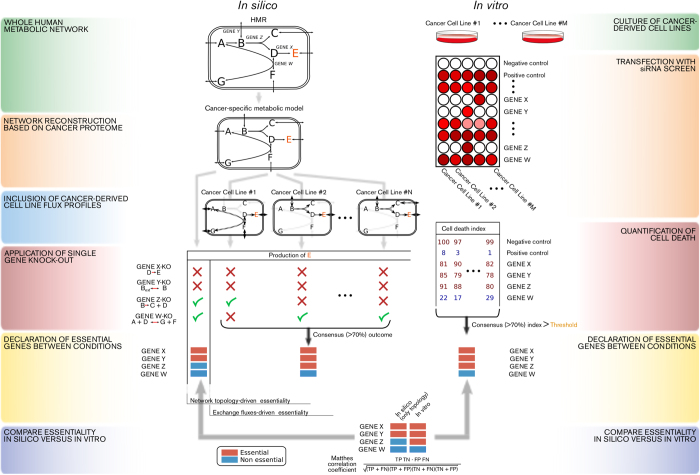
Strategy to measure the accuracy of flux balance analysis predictions of gene essentiality in cancer metabolism. Left part: the Human Metabolic Reaction (HMR) database was used as a generic genome-scale metabolic network to reconstruct a cancer-type specific network based on proteome data obtained from cancer specimen (in the example, the reaction *A ﬁ B* is absent in the cancer-specific model due to lack of the matched enzyme at the protein level for that cancer type). Successively, flux balance analysis is used to simulate whether a flux towards production of biomass (metabolite *E*) was feasible after every single gene-knockout, using as constraints either the topology of the cancer type-specific metabolic network or the measured fluxes for a number of exchange metabolites in different cancer type-derived cell lines. In the latter case a gene is deemed essential if it disables biomass production in ≥70% of the cell lines. Grey arrows indicate reactions not occurring in the network. Dashed arrows indicate measured fluxes in a cell line. Right part: cancer-derived cell lines were cultured and transfected with a library of siRNAs that target ~230 metabolic genes and cell number was determined after 4 days. If ≥70% of the cell lines passed a given threshold of cell death, the corresponding gene was deemed essential. Bottom: gene essentiality for the ~230 genes targeted by the siRNA library was compared *in silico* vs. *in vitro* and the accuracy of the predictions was calculated by several statistical measures (e.g. the Matthews Correlation Coefficients).

Flux balance analysis (FBA) is possibly the only modelling approach that has the potential to predict gene essentiality in cancer metabolism at the genome scale^39^. In this study, we sought to systematically validate whether FBA can be used to determine gene essentiality in cancer cell metabolism by comparing predictions with large-scale experimental datasets (Fig. 1). Therefore, the FBA problem was formulated to scan for a feasible flux distribution that enables the simultaneous biosynthesis of all human biomass components, the so-called biomass equation, in cancers growing in defined serum-containing medium^42^. In these conditions, the metabolic network is free to absorb any medium or serum metabolites (at any rate), which include sugars, amino acids, several metabolic intermediates and short chain fatty acids. In FBA, the emergence of a feasible flux distribution that can support biomass formation is generally limited by the introduction of constraints^43^,^44^ that can represent molecular or environmental limitations (e.g., the absence of a given enzyme in a cancer type or the unavailability of a nutrient in the microenvironment).

Here, we considered two typical sets of constraints: A) the topology of the cancer specific-metabolic network; and B) a profile of experimentally measured fluxes for a number of exchange metabolites (i.e., exchange fluxes) in a panel of cancer-specific cell lines (generally more than one cell line for each type of cancer). Using either of these two constraints we predicted gene essentiality using FBA by introducing a constraint that disables flux in the univocally encoded reaction(s). This constraint is commonly referred to as *in silico* single-gene knockout, and the gene is essential if the *in silico* single-gene knockout ablates biomass production. A gene knockout ablates biomass production if there is no flux distribution that allows the biomass equation to carry a flux, or if the knockout results in a substantial flux reduction. However, a gene knockout consents biomass production if there is no change in the flux through the biomass equation. Single-gene knockout resulting in no change in biomass production is mostly explained due to one of the following reasons: 1) gene redundancy, i.e., more than one gene encodes for the reaction(s) associated with the knockout; 2) pathway redundancy, i.e., there is an alternative pathway with the same overall stoichiometry that can compensate for the knockout; or 3) the reaction(s) encoded by the knocked-out gene are not active (dead end) at the studied condition. Depending on this outcome, a gene is declared essential or nonessential *in silico* for a certain cancer. If constraint B) is implemented, an *in silico* single-gene knockout may ablate or consent biomass production, depending on which profile of exchange fluxes is used as a constraint. In this case, the corresponding gene is declared essential *in silico* for the cancer type only if biomass production is ablated using exchange flux profiles from at least 70% of its corresponding cancer cell lines.

In principle, the proposed approach should capture all metabolic liabilities related to biomass formation induced by the network topology and to the activation of metabolic pathways induced by the exchange flux profile of a certain cancer. At the same time, it is noteworthy that the FBA problem formulated herein will not uncover other metabolic liabilities known to be associated with cancer survival, for example, maintenance of anti-oxidant pools^45^. To evaluate the gene essentiality predictions, we compared these to large-scale experimental data *in vitro*: in this case, a panel of cancer-specific cell lines derived from prostate adenocarcinoma (PC) or clear cell renal cell carcinoma (ccRCC), both cultured in defined serum-containing medium. The cells were transfected with a library of siRNA oligonucleotides that target approximately 230 metabolic genes. In the PC screen, induction of caspase activity was quantified after 96 h following transfection, whereas in the ccRCC screen, reduction in cell number was monitored. If at least 70% of the cancer cell lines passed a given threshold for caspase activity or cell number reduction, then the gene was declared essential *in vitro* for this cancer type (or nonessential *in vitro* if *vice versa*). The accuracy of the predictions was calculated using the Matthews correlation coefficient (MCC) and the related Fisher’s exact test statistics.

### Accuracy of flux balance analysis for gene essentiality in clear cell renal cell carcinoma metabolism

We decided to assess *in vitro* gene essentiality in the metabolism of ccRCC, as this is the most common form of kidney cancer^40^ and it exhibits a strong regulation and dependence on a reprogrammed metabolism following transformation^46^,^47^,^48^. Additionally, we have recently shown that it features a characteristically compromised metabolic network^41^. The reliance on specific metabolic reactions for survival suggests that this cancer may be particularly susceptible to disruptions in the metabolic network. A panel of 5 ccRCC cell lines (786-O, A498, 769-P, RCC4, and UMRC2) was transfected with a custom library of siRNA oligonucleotides targeting 230 different metabolic enzymes, transporters, and regulators involved in central carbon metabolism. For each siRNA, loss of viability was quantified by determining the mean cell number reduction relative to a negative control (non-targeting RISC-free) and a positive control (siRNA targeting ubiquitin B). The number of genes declared essential *in vitro* depends on the threshold chosen for the mean cell number reduction. We selected a 30% reduction for benchmarking purposes because the quantity of essential genes appears to reach a plateau at this value; note that no siRNA caused a cell number reduction greater than 50% (Supplementary Fig. 1). With this threshold, of the 217 tested siRNAs that overlap with the human metabolic network^49^, 20 gene knockdowns caused death in at least 70% (4 of 5) of the ccRCC cell lines and were thus deemed essential *in vitro* (Supplementary Fig. 2). In contrast, 136 tested siRNAs did not significantly affect cell number in at least 70% of the ccRCC cell lines and were conversely deemed nonessential *in vitro* (Supplementary Data 1). The remaining 61 genes were not classified, as their knockdowns had mixed effects across cell lines and therefore were not directly attributable to the ccRCC phenotype.

**Figure 2:**
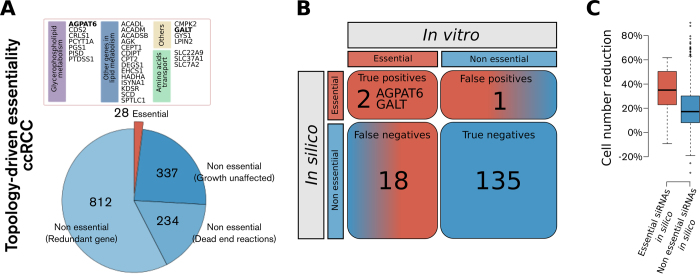
Gene essentiality in ccRCC metabolism as predicted by flux balance analysis using the metabolic network topology as a sole constraint for biomass formation positively compares to a functional RNAi screen targeting ~230 metabolic genes in a panel of ccRCC cell lines. **A**) Gene essentiality in ccRCC according to flux balance analysis using the metabolic network topology as only constraint for biomass formation. **B**) Contingency table for the comparison between the declaration of gene essentiality *in silico* vs. *in vitro* for those siRNAs in the library that had consensus effect in terms of cell number reduction in ≥70% of the cell lines. AGPAT6 and GALT are considered true positives (*p* = 0.04) because their ablation results in cell death *in silico* and *in vitro*. **C**) Boxplots of total cell number reduction for the groups of siRNAs predicted to be either essential (red) or non essential (blue) *in silico.*

Next, we predicted *in silico* gene essentiality using as the sole constraint the topology of the ccRCC metabolic network, as defined by a ccRCC genome-scale metabolic network^41^. We identified 28 essential genes and 1,383 nonessential genes (Fig. 2A). Topology-driven gene essentiality was found to be accurate at a statistically significant level (MCC = 0.226, *p* = 0.043, Fig. 2B). This approach detected two true positives (i.e., candidates essential both *in silico* and *in vitro*), namely *AGPAT6* and *GALT* (Fig. 2A); the expected number of true positives by chance is close to approximately zero ([TP] = 0.174). In this sense, we can assume that *AGPAT6* and *GALT* represent *bona fide* pivotal metabolic nodes in ccRCC, regardless of the exchange fluxes, which suggests that their essentiality is due to a loss of alternative redundant metabolic pathways or genes in ccRCC. Interestingly, siRNAs corresponding to genes predicted to be essential *in silico* result overall in a mean cell number reduction significantly higher than that for siRNAs corresponding to genes predicted not to be essential (*p* < 0.001, Wilcoxon rank-sum test, Fig. 2C).

**Figure 3:**
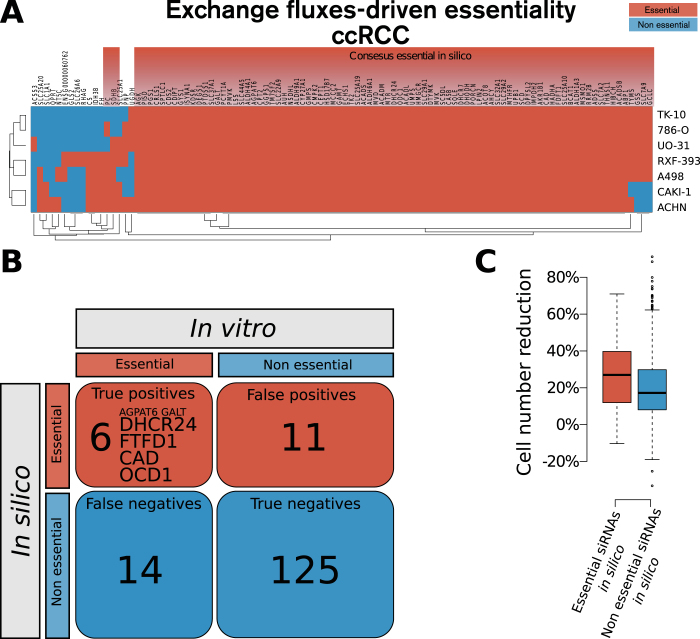
Gene essentiality in ccRCC metabolism as predicted by flux balance analysis using the profile of exchange fluxes from seven ccRCC cell lines in addition to the ccRCC network topology shows increased accuracy when compared with the RNAi screen. **A**) Gene essentiality in ccRCC according to flux balance analysis using the profile of exchange fluxes from seven ccRCC cell lines on top of ccRCC network topology. Each profile of exchange fluxes representing a ccRCC cell line entails a set of genes essential when using that profile. The heatmap features only genes that are essential using at least one flux profile. Finally, genes that are essential using at least 70% of the cell line flux profiles are deemed essential *in silico* in ccRCC. **B**) Contingency table for comparison between the declaration of gene essentiality *in silico* vs. *in vitro* for those siRNAs in the library that had consensus effect in terms of cell number reduction in ≥ 70% of the cell lines. Other than AGPAT6 and GALT, DHCR24, FTFD1, CAD, and OCD1 are true positives (*p* = 0.007). **C**) Boxplots of total cell number reduction for the groups of siRNAs predicted to be either essential (red) or non essential (blue) *in silico.*

Next, we also implemented exchange fluxes from a panel of seven ccRCC cell lines (786-O, A498, ACHN, CAK1-1, TK-10, RXF-393, and UO-31) as constraints^50^,^51^. Using this approach, eighty-seven genes were predicted to be essential in at least 70% (5 of 7) of the cell lines (Fig. 3A). When exchange fluxes were considered, the gene essentiality prediction was found to have an increased accuracy, when compared to the *in vitro* data (MCC = 0.235, *p* = 0.010, Fig. 3B). Additionally, in this case we observed a substantial mean cell number reduction for the group of siRNAs targeting genes predicted to be essential *in silico* compared to those predicted to be nonessential (*p* < 0.001, Fig. 3C). In particular, four additional genes were identified as true positives using this approach, namely *CAD*, *DHCR24*, *FDFT1*, and *ODC1* (Fig. 3A). It is likely that the essentiality of these genes is attributable to common metabolic requirements among ccRCC cell lines (e.g., a high lactate secretion to glucose uptake ratio or secretion of secondary metabolites), which induces dependence on the expression of enzymes that activate the related metabolic pathways. Interestingly, the accuracy of these predictions was not preserved if only exchange fluxes were considered, but the topology of the ccRCC metabolic network was neglected: we observed no significant predictive ability when the generic human metabolic network was used (MCC = 0.086, *p* = 0.339, Supplementary Fig. 3). The results of the accuracy achieved by FBA in these scenarios are reported in Table 1.

Taken together, these results suggest that in ccRCC metabolism, FBA is able to predict gene essentiality, although to a limited degree. Gene essentiality as exposed by FBA is in turn attributable to a rewiring of the metabolic network and exchange fluxes that contribute to biomass production. Conversely, it is conceivable that the 14 genes that were found to be essential *in vitro* but were not captured by FBA are essential because the gene products carry out metabolic tasks that are not ascribable to the biomass production simulated here. Alternatively, it also possible that redundant pathways available in the metabolic network are not active due to the presence of regulation *in vitro* or *in vivo* that is not considered in the FBA simulations, as suggested by studies of gene deletion in yeast^52^.

### Accuracy of flux balance analysis for gene essentiality in prostate adenocarcinoma metabolism

We next sought to define whether the accuracy of FBA predictions is cancer type-dependent. To this end, we used a published dataset that applied the same custom siRNA library in a panel of three prostate adenocarcinoma (PC) cell lines (LNcaP, PC3, DU145)^53^. Cell death was defined by induction of caspase activity, and we declared a gene essential *in vitro* if the corresponding siRNA caused apoptosis with a caspase activity *z*-score ≥ 2.5 (i.e., number of standard deviations from control) in at least 2 of the 3 cell lines, as adopted in the original study. Using these criteria, 14 metabolic genes were found to be essential in the PC cell lines (Supplementary Fig. 4). The topology of a PC specific metabolic network was reconstructed using the same pipeline followed to generate the previously employed ccRCC genome-scale metabolic model^41^ and was used as the sole constraint to perform FBA to predict *in silico* gene essentiality. We identified 37 essential genes, whereas 1,638 genes were classified as nonessential (Supplementary Fig. 5A). We also implemented exchange fluxes from a panel of two PC cell lines, PC3 and DU145, as constraints^50^,^51^, which resulted in the classification of 35 additional genes as essential in both these cell lines (Supplementary Fig. 5B).

Contrary to the results obtained for ccRCC, the accuracy of FBA predictions in PC was considerably lower when using metabolic network topology as the sole constraint (MCC = 0.082, *p* = 0.233, Supplementary Fig. 6A), and even worsened with the implementation of exchange fluxes (MCC = 0.039, *p* = 0.635, Supplementary Fig. 6C). However, when only topology was used, we observed a slightly higher mean caspase activity for the group of siRNAs targeting genes predicted to be essential *in silico* (*p* *=* 0.011, Supplementary Fig. 6B); this did not hold when exchange fluxes were also used as constraints (*p* = 0.152, Supplementary Fig. 6D). The results of the accuracy achieved by FBA in these scenarios are reported in Table 1. Interestingly, most genes predicted to be essential *in silico* participate in the biosynthesis of steroids. In particular, the two true positive genes detected by FBA, *MVD* and *NSDHL*, belong to this pathway. The inefficacy of exchange fluxes to unveil additional liabilities may be due to the low number of flux profiles available as constraints for PC (only 2 cell lines), as opposed to ccRCC (7 cell lines). However, it is also possible that the altered exchange fluxes in PC cells fuel pathways other than those required for biomass production and are therefore not captured by the FBA model used here. One of these pathways could indeed involve the synthesis of cholesterol for the production of steroid hormones, which play a major role in the development of PC^54^.

These results suggest that contrary to ccRCC, FBA fails to accomplish acceptable predictions of gene essentiality in PC metabolism. This may reflect the fact that PC cells are more robust in the task of synthesising biomass components. In support of this, Ros and colleagues identified a metabolic liability in PC that does not relate to biomass formation, but is involved in detoxification of reactive oxygen species (ROS)^53^. In addition, ccRCC metabolism could represent an ideal situation for the identification of metabolic liabilities using FBA because of its highly compromised metabolic network.

### Effect of the medium metabolites on the accuracy of flux balance analysis predictions

In FBA, the definition of metabolites available for uptake is a decisive constraint for the prediction of gene essentiality^43^. In simulations with microorganisms, the list of metabolites available for uptake mirrors the medium composition used in the controlled experimental setup. Because human cancer cell lines are normally cultured in serum-containing medium, the list of 150 metabolites adopted so far may potentially contain a large number of compounds that can be utilised *in silico* even though they are not utilised in any metabolic reactions by cells *in vitro* (e.g., bilirubin). To explore the extent to which the medium composition affects the accuracy of FBA predictions, we repeated all simulations using Ham’s medium, a nutrient poor medium adopted in previous studies to predict *in silico* gene essentiality of cancer cells^33^,^39^. This less permissive medium decreases the availability of alternative pathways. Thus, the number of essential genes predicted *in silico* increases for both ccRCC (Supplementary Fig. 7) and PC (Supplementary Fig. 8), when only the topology is used as a constraint and when exchange fluxes are also considered. However, these genes were mostly not found to be essential *in vitro*, and therefore the accuracy of the FBA predictions was lower for all four scenarios (Supplementary Fig. 9). We conclude that a broader definition of the medium improves FBA simulations in human systems and reduces the number of false negatives (i.e., genes essential *in silico* but not *in vitro*) induced by incorrect assumptions regarding the unavailability of certain metabolites to the cells. The results of the accuracy achieved by FBA in these scenarios are reported in Table 1.

### Effect of the choice for the cell death threshold *in vitro* on the accuracy of flux balance analysis predictions

Given that the definition of gene essentiality *in vitro* depends on the threshold selected for cell death (namely the mean cell number reduction in the ccRCC screen and the caspase activity *z*-score in the PC screen), we performed a sensitivity analysis on these thresholds for all tested scenarios (implemented constraints, cancer types, and medium definition). In the case of ccRCC, we observed a positive relationship between the accuracy of FBA predictions and the strictness of the definition of the threshold for cell death, at least up to the point where the number of essential genes *in vitro* is less than 10, which occurs for mean cell number reduction >40% (Supplementary Fig. 10). This trend was conserved in all scenarios, with the highest accuracy being achieved when using the topology of the ccRCC metabolic network in a serum-containing medium as the sole constraint to perform FBA; the lowest accuracy was observed when constraining the exchange fluxes in Ham’s medium. In the case of PC, the above trend was not observed for any scenarios (Supplementary Fig. 11). In particular, the accuracy of predictions was not noticeably different from a random predictor. Taken together, this indicates that the accuracy of *in silico* predictions is significant in ccRCC (but not in PC) for a reasonable range of thresholds upon which a gene is declared essential *in vitro*.

### Effect of cancer cell line exchange fluxes on the inference of gene essentiality in a certain cancer

**Figure 4:**
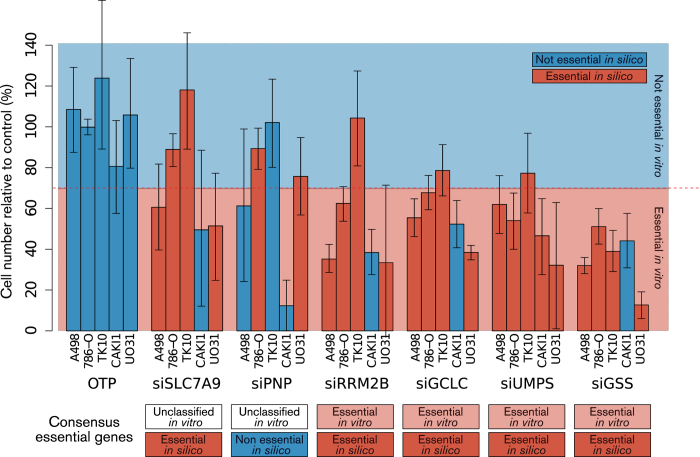
Validation of predicted gene essentiality in ccRCC. Five ccRCC cell lines that match the flux profile constraints implemented to predict gene essentiality in ccRCC were transfected with siRNA targeting SLC7A9, PNP, RRM2B, GCLC, UMPS and GSS. A non-targeting oligonucleotide, OTP (scrambled siRNA), was used as negative control. . Each bar represents the mean cell number reduction relative to control together with the 95% highest density interval of two experiments performed in triplicate. The consensus outcome across cell lines in terms of gene essentiality is shown below each set of bars corresponding to a certain silenced gene. Genes that, if silenced, cause a ≥ 30% reduction in cell number relative to the non-targeting RISC-free siRNA in ≥ 70% of ccRCC cell lines are deemed essential *in vitro.* The consensus outcome for each silenced gene is compared to the prediction of essentiality *in silico* for the corresponding gene in ccRCC (compare with Fig. 3A).

Because FBA proved powerful in exposing the metabolic liabilities of ccRCC, we decided to validate some of the *in silico* predictions of gene essentiality. In particular, we tested the extent to which exchange fluxes from ccRCC cell lines can be used to infer gene essentiality attributable to the ccRCC phenotype. To this end, we selected some genes that were differentially classified as essential depending on the cell line flux profile, but still classified as essential in ccRCC according to a consensus outcome, i.e., essential in >70% of cell lines. We chose to test the predictions for *GCLC*, *GSS*, *SLC7A9* (considered essential *in silico* for ccRCC because they were classified as such in 5 of the 7 cell lines), and *PNP* (considered nonessential *in silico* for ccRCC because it was classified as such in 3 of the 7 cell lines). In addition, we tested *UMPS* and *RRM2B*, which were deemed essential *in silico* for all ccRCC cell lines upon implementation of every exchange flux profile. Next, the corresponding genes were silenced in five of the seven cell lines whose exchange fluxes were used to constrain the FBA predictions (786-O, A498, CAKI-1, TK10, and UO31). In accordance with the threshold for cell death adopted above, a gene was declared essential *in vitro* for ccRCC if more than 70% of cell lines tested (e.g., at least 4 of 5) exhibited at least 30% mean cell number reduction compared to control (Fig. 4).

At the level of gene essentiality in ccRCC, the consensus predictions for *RRM2B, GCLC*, *UMPS*, and *GSS* were confirmed *in vitro.* However, *PNP* and *SLC7A9* knockouts showed mixed effects across cell lines *in vitro*. Hence, the essentiality of these genes in ccRCC could not be inferred from this experiment. Overall, this result suggests that ccRCC cell line exchange fluxes can entail some common metabolic requirements associated with the ccRCC phenotype, which can be exploited to predict gene essentiality in ccRCC metabolism. However, the exchange flux measurements appear to be insufficient *per se* to achieve reliable predictions for a specific cell line. Indeed, we observe that only 17 of the 30 individual predictions were replicated *in vitro* if the cell-line-specific exchange fluxes were used for the prediction of essentiality for the corresponding cell line.

### Characterisation of gene essentiality in ccRCC metabolism

**Figure 5:**
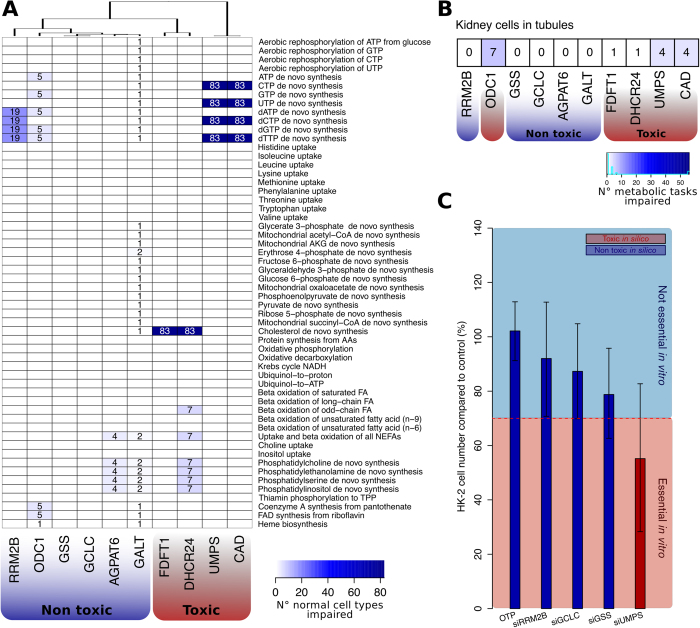
In silico toxicity for the knockouts in normal cells of genes essential in ccRCC. **A**) Number of normal cell types where a certain metabolic task is impaired upon *in silico* knockout. For each of the ten genes found essential in ccRCC according to this study (*RRM2B, ODC1, GSS, GCLC, AGPAT6, GALT, FDFT1, DHCR24, UMPS,* and *CAD*; columns), it was tested if the corresponding *in silico* gene knockout affects the feasibility of 56 different metabolic tasks (rows) in 83 genome-scale metabolic models representing normal, non-tumourigenic cell types. The numbers within the heatmap indicate how many of normal cell types (out of the 83) showed a certain metabolic task that was no more feasible upon the knockout (white cells indicate that none of the cell lines showed an effect). The knockout of *AGPAT6, GALT, GCLC, GSS, ODC1*, and *RRM2B* did not impair more than 50% of normal cell types and are hence considered non-toxic to normal cells. On the contrary, *FDFT1, DHCR24, UMPS* and *CAD* knockouts affected some essential metabolic tasks in all normal cell types and are thereby considered toxic to normal cells. **B**) Number of metabolic tasks impaired upon *in silico* knockouts in a kidney cell in tubule model. In addition to *FDFT1, DHCR24, UMPS* and *CAD, OCD1* knockout is predicted to be toxic because it disables seven metabolic tasks. On the other hand, knockouts of *AGPAT6, GALT, GCLC, GSS,* and *RRM2B* are predicted to be non-toxic and these genes are thus considered selectively essential in ccRCC. **C**) Validation of toxicity for *GCLC, GSS, RRM2B* and *UMPS* knockouts in a normal kidney epithelial cell line, HK-2. Cells were transfected with siRNA targeting RRM2B, GCLC, UMPS and GSS and a non-targeting scrambled siRNA, OTP, was used as negative control. Each bar represents the mean cell number reduction relative to control together with the 95% highest density interval of two experiments performed in triplicate. In line with the predictions, *UMPS* knockout caused a substantial cell number reduction in HK-2 cells compared to knockouts of *GCLC, GSS,* and *RRM2B*, thereby indicating a substantially superior toxicity in normal kidney epithelial cells.

FBA exposed some metabolic liabilities in ccRCC that are unlikely to have been predicted by chance. Therefore, we sought to characterise those genes that were classified in this study as essential *in silico* and validated *in vitro.* This list includes ten metabolic genes: *AGPAT6, CAD*, *DHCR24*, *FDFT1*, *GALT, GCLC, GSS, ODC1, RRM2B,* and *UMPS.* First, we predicted whether these gene knockouts would be toxic for the execution of essential metabolic functions, i.e., whether the *in silico* gene knockouts compromise the metabolism of normal cell types. As previously described^33^, we simulated the essentiality of these genes in 83 normal cell types by checking whether 56 primary metabolic tasks (e.g., synthesis of cholesterol or oxidative phosphorylation) could be carried out *in silico* upon application of the corresponding *in silico* gene knockout. In all normal cell types, the simulation revealed that knockout of *CAD* or *UMPS* ablates the *de novo* biosynthesis of pyrimidines, while *FDFT1* and *DHCR24* knockouts impede the production of cholesterol in normal human cell types (Fig. 5A). However, the remaining 6 genes had only minor toxic effects (in < 50% of cell types), and can thus be regarded as nontoxic to normal cells.

Next, we specifically checked the toxicity of these gene knockouts in tubular kidney cells, where ccRCC is thought to originate from^55^. In this case, the *in silico* knockout of *ODC1* was found to be toxic because it impaired seven essential metabolic tasks in normal kidney cells. On the contrary, *AGPAT6, GALT, GCLC, GSS*, and *RRM2B* knockouts did not compromise any metabolic task and can thus be considered as selectively essential in ccRCC (Fig. 5B). To test the quality of these predictions, we ablated *GCLC, GSS*, *RRM2B*, and *UMPS* in an immortalised, non-tumourigenic kidney epithelial cell line (HK-2) using RNAi. These four genes were not part of the siRNA screening library but were predicted by FBA to be essential both *in silico* and *in vitro*. In accordance with the *in silico* predictions of toxicity, we observed cell death when *UMPS* was knocked out in HK-2 cells, while *GCLC, GSS*, and *RRM2B* knockouts caused a minor cell number reduction, above the adopted threshold for cell death (Fig. 5C).

**Figure 6:**
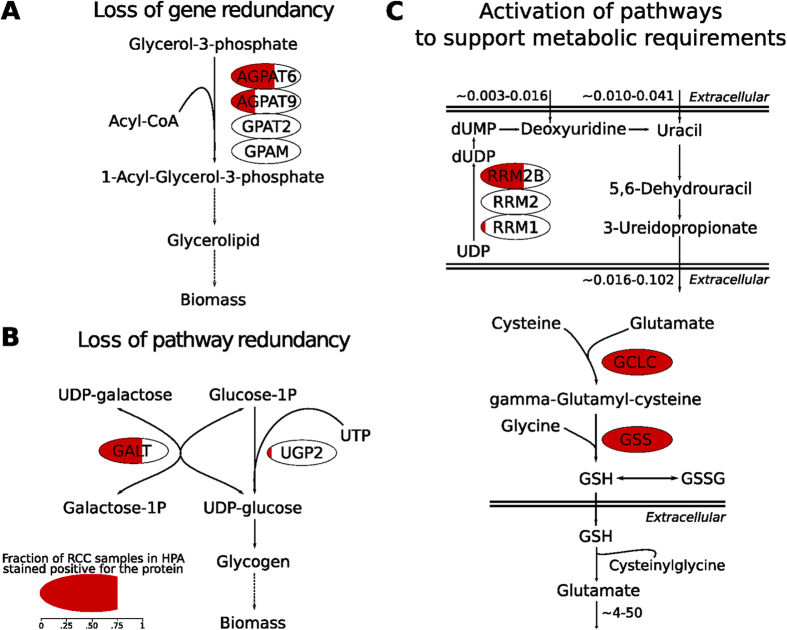
In silico elucidation of the mechanisms of essentiality for the five genes selectively essential in ccRCC. **A**) *AGPAT6* is essential only in ccRCC because of loss of gene redundancy. In ccRCC, the repression of *AGPAT9*, *GPAT2*, and *GPAM* in glycerolipid metabolism renders the pathway solely dependent on AGPAT6 to produce essential lipids for biomass. **B**) *GALT* is selectively essential because of loss of pathway redundancy in ccRCC. Low or no expression of UGP2 forces the flux through GALT to produce glycogen in ccRCC. **C**) *RRM2B, GCLC* and *GSS* are essential only in ccRCC because of specific metabolic requirements of ccRCC cells that activate the corresponding pathway (flux rates are shown in fmol cell^−1^ h^−1^). Top: the measured secretion rate of 3-ureidopropionate in ccRCC cell lines is not matched by the observed uptake rate of its direct precursors, uracil and deoxyuridine. This forces a flux active in the catabolism of UDP (part of the pyrimidine degradation pathway) to compensate for the observed 3-ureidopropionate secretion rate. One of the pathway steps is uniquely catalyzed by RRM2B, given that the other genes associated to this reaction (RRM1 and RRM2) are not expressed in ccRCC. Bottom: ccRCC cell lines secrete glutamate at a high rate and the only flux distribution that fits glutamate secretion in the ccRCC metabolic network requires the cleavage of extracellular glutathione (GSH). Extracellular GSH is in turn derived from *de novo* GSH intracellular synthesis that is catalyzed by GSS and GCLC. Noteworthy, the reduction of reactive oxygen species like H_2_O_2_ by GSH is a metabolic function preserved in the predicted flux distribution. For each protein, the red shading represents the fraction of ccRCC samples in which the protein is expressed according to the Human Protein Atlas.

Subsequently, we attempted to elucidate the putative mechanisms at the flux level underlying the essentiality of the *AGPAT6, GALT, GCLC, GSS*, and *RRM2B* genes, which were predicted to be toxic to none or only to a few of the normal human cell types, and in particular were predicted to be nontoxic to tubular kidney cells. *AGPAT6* and *GALT* were found to be essential when using the topology of the ccRCC metabolic network as the sole constraint for FBA, which is indicative of a loss of pathway redundancy in key steps involved in biomass synthesis. In the human metabolic network, the *AGPAT6*-encoded reaction, i.e., the conversion of glycerol-3-phosphate to 1-acyl-glycerol-3-phosphate, is associated with additional isoenzymes, *AGPAT9, GPAT2,* and *GPAM*. However, according to the Human Protein Atlas, which supported the reconstruction of the ccRCC metabolic network, *AGPAT6* is the only member of the family of lysophosphatidic acid acyltransferase genes appreciably expressed in ccRCC^56^. Therefore, when *AGPAT6* is knocked out, the production of glycerolipids, which is required for biomass production, becomes unfeasible (Fig. 6A), making *AGPAT6* an essential gene in ccRCC.

Regarding GALT, this enzyme is pivotal in the ccRCC metabolic network because it catalyses the second step of the Leloir pathway of galactose metabolism (conversion of UDP-galactose to UDP-glucose). Examination of the flux space in ccRCC revealed that this reaction fuels the production of UDP-glucose, which is needed for the biosynthesis of glycogen. Knockout of *GALT* thus results in growth ablation due to the inability to produce glycogen, here considered to be an essential biomass component. This pathway can be bypassed via UGP2, which condenses glucose-1-phosphate with UTP to yield UDP-glucose, but *UGP2* is not expressed in ccRCC (Fig. 6B). The essentiality of GALT in ccRCC is determined by the inactivity of this parallel pathway; this represents an example of loss of redundancy within the topology of the metabolic network.

The three additional genes, *GCLC, GSS*, and *RRM2B*, were classified as essential only when constraints on exchange fluxes were implemented. Their essentiality is likely to be due to a loss of redundancy in the ccRCC network when metabolite fluxes are constrained by measured uptake and secretion rates. To explore how the fluxes were distributed before the implementation of the exchange fluxes, we relieved each of these constraints one at the time until biomass production was restored, thereby allowing us to associate gene essentiality with a particular exchange flux. We found that *RRM2B* is associated with the flux of 3-ureidopropionate, a product of the uracil degradation pathway, which is secreted by all ccRCC cell lines (range: 0.016 to 0.102 fmol cell^−1^ h^−1^) (Fig. 6C, left). This secretion rate is not matched by the uptake rate of either of its two precursors, uracil and deoxyuridine (range: 0.003 to 0.016 and 0.010 to 0.041 fmol cell^−1^ h^−1^, respectively). Thus, it is necessary for cells to activate a flux to degrade UDP to sustain the given 3-ureidopropionate secretion rate, and one of these steps is catalysed by RRM2B. According to the Human Protein Atlas, the two other genes associated with this step (namely *RRM1* and *RRM2*) are not expressed in ccRCC, and thus they cannot compensate for this flux if *RRM2B* is knocked out, making *RRM2B* essential.

In the case of *GCLC* and *GSS*, the essentiality is associated with the secretion of glutamate, which occurs at remarkably high rates (approximately 4 to 50 fmol cell^−1^ h^−1^) in ccRCC cell lines. The analysis of the ccRCC flux space unveiled that elevated rates of extracellular glutamate accumulation derive from the catabolism of extracellular glutathione (GSH) carried out by different gamma-glutamyl transferases. Indeed, the reconstructed ccRCC metabolic network does not include alternative pathways that support the secretion of glutamate, such as the x_C_^–^ system. Despite the evidence that the x_C_^–^ system, an antiporter responsible for cystine uptake via a 1:1 exchange with glutamate, is expressed in the kidney^57^, no evidence for the encoding gene, *SLC7A11*, is reported at the protein level by the Human Protein Atlas; it was therefore not included in the reconstructed network. In the absence of alternative pathways, the only flux distribution returned by FBA that fits glutamate secretion requires the cleavage of extracellular GSH. This is in turn dependent on the secretion of *de novo* synthesised intracellular GSH, which is catalysed by GSS and GCLC (Fig. 6D, right). The genes are therefore classified as essential to support this flux distribution. At the same time, GSH is also utilised to reduce peroxides/reactive oxygen species (ROS). In this process, GSH is oxidised and dimerises with another moiety to form GSSG, which can be catalytically recycled to GSH. Therefore, this flux distribution also includes the expected role of GSH in ROS detoxification. Overall, FBA was able to predict a model that associates the essentiality of *GSS* and *GCLC* to the observed secretion of glutamate. Nevertheless, we acknowledge that model incompleteness (attributable to a lack of functional gene annotation in the metabolic network, as in the case of *SLC7A11* in ccRCC) may be a factor that affects the reliability of this prediction, as recognised in earlier works on FBA predicted gene essentiality^58^,^59^.

## Discussion

In the last decade, increasing evidence supports the notion that cancer cells reprogram their metabolism and are therefore susceptible to disruption of the metabolic network^3^. Despite the promise that flux balance analysis (FBA) enables prediction of gene essentiality in cancer metabolism at the genome scale^21^, we have observed a scarcity of methodical studies that assess these potential benefits critically. Considering the widespread use of FBA in the systems biology community^60^, we applied fundamental principles of FBA to measure the accuracy of the predictions against large-scale gene essentiality experiments performed *in vitro*. We evaluated the efficacy of this method for two cancer types, clear cell renal cell carcinoma (ccRCC) and prostate adenocarcinoma (PC), for a variety of parameters: types of FBA constraints used, complexity of the *in silico* medium (i.e., the spectrum of metabolites available for uptake), and the numerical threshold for cell death applied to the *in vitro* experiments. A summary of the accuracy for all tested scenarios is given in Table 1.

Our findings suggest that FBA is sufficiently accurate to expose metabolic liabilities in ccRCC. The highest accuracy was achieved by FBA using a ccRCC-specific metabolic network that was further constrained by exchange fluxes determined experimentally across a panel of seven ccRCC cell lines and using an *in silico* serum-containing medium, which allows the flexible uptake of 150 different metabolites (Matthews Correlation Coefficient = 0.235). The accuracy improved with stricter definitions of the threshold for cell death *in vitro,* whereas it worsened when a more restricted medium metabolite composition, as found in Ham’s medium, was applied *in silico.* This medium composition is not representative of the actual culture conditions used for the *in vitro* experiments, but had previously been used in similar studies^39^,^61^. However, FBA was found not to be predictive for gene essentialities in PC, where the accuracy was not better than a random predictor (MCC = 0.039, for the same scenario described above).

In general, poor predictions may be ascribed to different assumptions beyond FBA. First, we considered gene essentialities in metabolism based on the ability to carry flux towards biomass formation. Although this is an undisputable requirement for cancer cell proliferation, there is evidence that survival of cancer cells also depends on other metabolic functions, most notably NADPH production and anti-oxidant synthesis^45^,^62^. Second, we also classify a gene as essential if the *in silico* knockout cannot satisfy certain cancer type-specific metabolic requirements, here represented by the profiles of exchange fluxes in several cancer type-specific cell lines (one such requirement could be lactate secretion in a specified range of rates). The number of profiles available for a cancer type may affect the specificity of certain metabolic requirements (for PC, only two profiles of exchange fluxes were available), therefore biasing the quality of the *in silico* predictions when these are enforced as constraints in FBA. Third, we disregarded enzyme complexes because current human genome-scale metabolic models do not report such annotation systematically^63^,^64^. Hence, if more than one enzyme is associated to a reaction in the model, all genes encoding for these enzymes are automatically excluded from the *in silico* single-gene knockout and classified as redundant. Furthermore, genome-scale metabolic models such as those used for this study represent the best models to our knowledge in terms of metabolic reactions occurring in a cell, but model incompleteness is known to affect *in silico* predictions of essential metabolic genes^59^. Finally, the evaluation of the accuracy of the *in silico* prediction is also affected by the accuracy of the *in vitro* experiments. Indeed, metabolic screens using siRNA libraries may produce false negatives due to insufficient silencing and are liable to significant off-target effects that can both positively and negatively affect the viability of transfected cells.

As part of this evaluation, FBA unveiled the inherent fragility of ccRCC metabolic processes that contribute to biomass growth or support certain metabolic requirements. A number of recent studies have evidenced the centrality of metabolism in ccRCC^41^,^46^,^47^, and these findings further support the notion that ccRCC is dependent on specific metabolic genes to sustain proliferation. At the same time, our work leverages on a metabolic model for ccRCC and cultured ccRCC cell lines to assess vulnerabilities for this disease. Hence, this approach cannot likely capture the genomic diversity and complexity of ccRCC^65^,^66^. However, it should prove useful to expose metabolic liabilities that are transversal to the ccRCC phenotype. In this context, we describe five genes, *AGPAT6*, *GALT, GCLC, GSS*, and *RRM2B,* which are essential to ccRCC but are potentially dispensable in normal cell types. In addition, FBA can also be used to explore the mechanisms that render a gene essential *in silico*.

One of the mechanisms by which this essentiality arises is loss of gene redundancy. AGPAT6 is the only expressed enzyme isoform that can commit glycerol-3-phosphate into glycerolipid biosynthesis. Thus, glycerolipid synthesis is clearly a sensitive pathway in ccRCC, perhaps exacerbated by the lack of expression of enzymes within alternative routes due to a loss of heterozygosity in the corresponding gene loci, as recently suggested^41^. Interestingly, some members of the AGPAT family may have a causal role in cancer development^67^, and further work is required to elucidate the roles of these genes in ccRCC. A second possible mechanism is loss of pathway redundancy, resulting in enhanced dependence on the remaining reactions. This mechanism of gene essentiality revealed by FBA in ccRCC is exemplified by GALT, a component of the Leloir pathway. Downregulation of the enzymes of an alternative pathway in ccRCC induced dependence on *GALT* expression. In accordance with the findings of our simulations, there is evidence that *UGP2* and *GALT* homologs provide redundancy for this pathway in yeast^68^. Moreover, it is has been shown that *GALT*-deficient mice can sustain glycogen synthesis through the pathway branch catalysed by the murine homolog of *UGP2*^69^, thus supporting the predicted mechanism that confers essentiality to *GALT* in ccRCC. The pivotal role of GALT in glycogen biosynthesis may in addition underscore the typical phenotype of ccRCC cells, which are characterised by high levels of glycogen accumulation. Finally, FBA using flux rates as additional constraints allowed us to identify gene essentialities associated with specific exchange fluxes in ccRCC cell lines. The essentiality of *GCLC* and *GSS* was linked to the secretion of glutamate, whereas the essentiality of *RRM2B*, an enzyme in deoxyribonucleotide metabolism, was linked to the secretion of 3-ureidopropionate. *GCLC* and *GSS* play a fundamental role in the intracellular detoxification of ROS by catalysing two successive steps in the biosynthesis of glutathione^70^. This biological process plays a prominent role in carcinogenesis^71^ and has been postulated to be of central importance in the rewiring of cancer metabolism^45^. Therefore, it is likely that the essentiality of *GCLC* and *GSS* in the *in vitro* experiments stems from their functions in the control of intracellular ROS levels.

Here, we find that *de novo* synthesis of GSH is also associated with glutamate secretion in the absence of other systems that can fulfil this function in ccRCC. Although this may be an artefact due to model incompleteness, we show that GCLC-GSS can sustain a flux distribution in which extracellular GSH is catabolised into cysteinylglycine and glutamate, therefore explaining the observed glutamate secretion in ccRCC cell lines. In a similar fashion, the consistent secretion of 3-ureidopropionate observed in ccRCC cell lines combined with an unmatched uptake rate of its direct precursors implies that *RRM2B* is active in supporting uridine-derived 3-ureidopropionate. *RRM2B* exerts its function in deoxyribonucleoside biosynthesis and in DNA damage repair, and in this role it appears to hinder cancer progression^72^,^73^,^74^,^75^. Nevertheless, *RRM2B* function in ccRCC may be different given the lack of expression of *RRM1* and *RRM2* for supporting nucleotide biosynthesis. Indeed, this pathway was found to be compromised uniquely in ccRCC compared to four other cancer types^41^.

In conclusion, in this study we show the strength and limitations of FBA for the prediction of gene essentiality at a genome scale in cancer metabolism. In addition, we report five metabolic genes selectively essential in a particular cancer type, i.e., ccRCC. Importantly, FBA can be used to identify potential mechanisms by which these gene essentialities arise and thereby provide testable hypotheses. We argue that accounting for metabolic liabilities other than biomass generation and the integration of additional layers of high-throughput data may lead to an even more complete description of the essentiality landscape in cancer metabolism.

## Materials and Methods

### Cell culture and reagents

786-O, 769-P, A498, CAKI-1, RCC4, TK10, UMRC2, and UO31 clear cell renal cell carcinoma (ccRCC) cell lines were maintained (and transfected) in DMEM supplemented with 4.5 g/l D-Glucose, 0.11 g/l Sodium Pyruvate (Gibco), 4 mM Glutamine (CRUK, Clare Hall, Cell Services), 100 Units/ml Penicillin / 100 ug/ml Streptomycin (Gibco) and 10% Fetal Bovine Serum (Gibco). For the RNAi screen, RCC4, UMRC2, A498, 786-O and 769-P cells were transfected in triplicates in a 96-well format with 37.5 nM siRNA SMARTpools (Dharmacon siGENOME) targeting the genes of interest using Dharmafect2 as transfection reagent. For the validation experiment for *GCLC, GSS, PNP, RRM2B, SLC7A9,* and *UMPS*, 786-O, A498, CAKI-1, TK10, and UO31 cells were transfected in two independent experiments, triplicates each in a 96-well format with 37.5 nM siRNA SMARTpools (Dharmacon siGENOME) targeting the genes of interest using Dharmafect2 as transfection reagent. For the validation experiment for *GCLC, GSS, RRM2B,* and *UMPS*, HK-2 cells were transfected in two independent experiments, triplicates each in a 96-well format with 37.5 nM siRNA SMARTpools (Dharmacon siGENOME) targeting the genes of interest using Dharmafect2 as transfection reagent. In all cases, after 96 h (with a media top up after 24 h), cells were fixed in 80% ethanol over night and subsequently stained with DAPI (Sigma). Cell number was determined using an ACUMEN ^e^X3 laser-scanning fluorescent micro plate cytometer. For the purpose of data normalization, the non-targeting RISC-free transfection was used as negative control, while ubiquitin B (*UBB*) and polo-like kinase 1 (PLK1) served as positive killing control.

### Quantification of cell death

In the case of ccRCC, cell death was quantified in terms of reduction of cell number upon siRNA transfection in cells scaled to the effect of the negative and positive controls. For each replicate, 9 positive killing controls (*UBB*) and 12 negative controls (RISC-free) were transfected. In a given cell line *c* for a given replicate *r*, the cell number reduction caused by siRNA *s* was linearly interpolated as in equation (1):


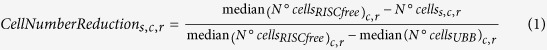


Then for each cell line, the mean cell number reduction is computed as the average across replicates. We declare a gene essential *in vitro* in ccRCC if the mean cell number reduction upon transfection of the corresponding siRNA for at least 70% of the tested cell lines is above 30%. For all these genes, we verified that the associated mean cell number reduction is statistically significantly greater than 0 (one sided *t-*test, *p* < 0.05). This data is collected in Supplementary Data 1. In the case of PC, processed data containing caspase activity *z*-score was retrieved from the study^53^. We declare a gene essential *in vitro* in PC if the caspase activity *z*-score upon transfection of the corresponding siRNA for at least 2 of the 3 tested cell lines is above 2.5, as adopted in the original study.

### Statistical tests

The Fisher’s Exact Test was carried out using the total number of tested siRNA in the library that could be compared to the *in silico* single gene-knockout test as the universe and it was performed in R. The 95% highest density interval (HDI) for cell number variation relative to RISC-free control was calculated by Bayesian estimation under the following assumptions: data are sampled from a *t-*distribution of unknown and to be estimated normality (i.e. degrees of freedom); high uncertainty on the prior distributions; the marginal distribution is well approximated by a Markov chain Monte Carlo sampling with no thinning and chain length equal to 100’000. The estimation was performed using the BEST R-package^76^ (the above assumptions are reflected by the default parameters).

### Flux balance analysis

The ccRCC genome-scale metabolic model (iRenalCancer1410) was downloaded at www.metabolicatlas.org. The PC genome-scale metabolic model (iProstateCancer1675) was reconstructed using the same pipeline as for iRenalCancer1410^41^. The models are inherently mass and charge balanced, and reaction directionalities to reflect thermodynamic constraints were not modified. Apart from changing a misannotated reaction incorrectly associated with *OGDH* to its correct associated gene, *ODC1,* no other modifications to the models were operated. The lists of metabolites available for uptake or secretion in the serum-containing medium (FBS) or in Ham’s medium are given in Supplementary Data 2. For FBS, this list was compiled by merging the list of metabolites exchanged in cell line cultures growing in fetal bovine serum medium according to Jain and coworkers^51^ and other compounds known to be present in this medium^42^. In the case of ccRCC, 94 metabolites could be matched with a preexisting uptake reaction in the network, 6 metabolites are present in the extracellular compartment but did not have a preexisting uptake reaction, and 51 metabolites are only present in the cytosolic compartment. We added an exchange reaction for each of the latter 57 metabolites, modeled as energy-free diffusion (i.e. => metA[s/c]). In the case of PC, 92 metabolites could be matched with a preexisting uptake reaction in the network, 7 metabolites are present in the extracellular compartment but did not have a preexisting uptake reaction, and 46 metabolites are only present in the cytosolic compartment. We added an exchange reaction for each of the latter 53 metabolites, modeled as energy-free diffusion (i.e. => metA[s/c]). For Ham’s medium, the list was retrieved from^33^. In both the case of ccRCC and PC, 38 metabolites could be matched with a preexisting uptake reaction in the network, 1 metabolite is present in the extracellular compartment but did not have a preexisting uptake reaction, and 4 metabolites are only present in the cytosolic compartment. We added an exchange reaction for each of the latter 5 metabolites, modeled as energy-free diffusion (i.e. => metA[s/c]). Unless cell line specific exchange fluxes were used, all the above exchange reaction can span any real value from –1000 to +1000, while any other exchange reaction was bounded to 0 for uptake. It should be noticed that this is a critical step for the simulations which follow: the possibility to freely exchange these metabolites allows to take in account all the extremely different metabolic states that a cell may adopt in response to the availability of these metabolites (e.g. lactate may be either secreted as a by-product of glycolysis or absorbed and catabolized as a carbon source). For both ccRCC and PC, the used biomass equation is the built-in reaction in iRenalCancer1410, which was in turn adapted from^35^. This reaction accounts for all major macromolecular components in the biomass (e.g., membrane lipids, proteins, etc.), and the respective stoichiometric coefficient is reflective of the contribution of each component in 1g of cancer biomass. The simulation of a single gene knockout using FBA was performed by formulating the linear program problem (2) for each gene *g* in the model:


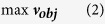



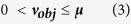



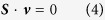



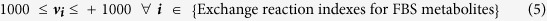



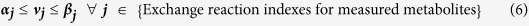



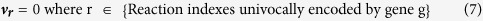


where *v*_*obj*_ is the flux through the biomass equation, 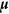 is the experimental growth rate (for simulation using cell line specific fluxes) or an arbitrary number (for simulation without specific constraints on the exchange reaction), ***S*** is the stoichiometric matrix of the model (that is a *m* x *n* matrix where *m* is the number of metabolites and *n* is the number of reactions and each (*i,j*) entry is the stoichiometric coefficient of the metabolite corresponding to row *i* in the reaction corresponding to column *j*), ***v***is the vector containing the values of the fluxes through each reaction in the model, and α (resp. β) are the lower (resp. upper) bound for the exchange flux corresponding to each metabolite measured in^51^ and adjusted by^50^. These bounds were implemented only in the simulations using cell line specific fluxes. For a given cell line, they were calculated for each measured metabolite *j* as 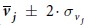, where 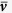
and 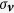 are the mean and the standard deviation of the corresponding exchange flux in the two replicate measurements. In the simulations using cell line specific exchange fluxes, the biomass equation coefficients were multiplied by a conversion factor equal to 550 pg_DW_ cell^−1^ to accommodate the fact that exchange fluxes were measured in fmol cell^−1^ h^−1^ instead of mmol g_DW_^−1^ h^−1^ as normally assumed in genome-scale metabolic modeling^77^.

The problem was formulated using native functions in the RAVEN Toolbox^78^ and solved using MOSEK v.7. Simulation results are reported in Supplementary Data 3. All constrained simulation-ready models are available through the website http://www.metabolicatlas.com/.

Importantly, for the purpose of the study, the optimization part is not relevant. Indeed, we are interested on whether a feasible region exists upon the constraint imposed by the gene knockout (7), i.e. whether the fact that the encoded reactions cannot carry flux implies no flux in the biomass equation. However, if also exchange fluxes were implemented to perform FBA (6), we took in account a significant reduction (min. 50%) of the optimum (which is upper bounded by the experimental growth rate) to classify a gene as essential *in silico*. Besides this, a gene is deemed essential *in silico* when there is no solution to (2), i.e. there cannot be found a flux distribution such that the biomass equation carries flux. This is indeed valuable in light of the consideration above: among all the available metabolic states permitted by the availability of serum metabolites, no scenario allows for a flux towards all biomass precursors simultaneously.

It should be noticed that, when implementing cell line specific exchange fluxes, a set of numerical constraints had to be neglected and converted to ±1000. This operation is obligated by the fact that some measured fluxes are not consistent with the network topology. Thus, either the measured compounds are not used by the cellular reaction network or further experimental validation is required. It may also be that the model is not complete and it should be updated in an iterative fashion, a process normally encountered in genome-scale models^79^. Examples of these inconsistencies are the conjugated bile acids (such as glycochenodeoxycholate) or anthranilate, that were measured to be absorbed by the cancer cell lines in Jain *et al.* study^51^, yet there is no evidence that these compounds can be degraded in any metabolic reaction accounted in the model. In other cases, coupled measured fluxes are stoichiometrically unbalanced. To get the minimum set of constraints (6) that had to be lifted to get a feasible solution to the problem (2) (neglecting the constraint 7), we formulated a linear program that iteratively searches for the minimum sum of the fluxes that must be supplemented to each exchange flux in (6) such that all other constraints are satisfied while *v*_obj_ > 0. In the end, all exchange fluxes in (6) that required a supplementary flux (i.e. the imposed bounds in the original problem would be infeasible) were instead bounded to ±1000. This procedure had been repeated for each cell line specific to a certain cancer type, and for all successive simulations, only the exchange fluxes that were feasible in all cell lines for a given cancer were retained. In ccRCC, 61 and 38 exchange fluxes were coordinately bounded for all seven ccRCC cell lines in FBS and Ham’s medium respectively (Supplementary Fig. 12–15). In PC, 60 and 57 exchange fluxes were coordinately bounded for all two PC cell lines in FBS and Ham’s medium respectively (Supplementary Fig. 16–19).

### Characterization of in silico essentiality

The test for toxicity in normal cell types was performed as previously described^33^. Briefly, 83 normal cell type genome-scale metabolic models were downloaded at www.metabolicatlas.org. For each model, a list of 56 metabolic tasks was simulated under the constraint (7) for each gene classified as essential *in silico* and validated as such *in vitro.* If no solution were found, the gene knockout is deemed toxic for a certain normal cell type. If more than 50% of the 83 normal cell types show no toxicity in any metabolic task upon knockout of a gene essential in a cancer, than the gene is regarded non toxic to normal cells. Furthermore, if knockout of a gene essential in a cancer shows no toxicity in any metabolic task in the supposed cell type of origin, than the gene is regarded selectively essential to a cancer.

To elucidate the mechanism through which a gene is selectively essential *in silico*, different simulations were carried out according to the constraint that first resulted in an unfeasible solution:

1. In the case of genes essential using as a sole constraint to perform FBA the topology of a cancer metabolic network, there are two possible explanations. In the first scenario, a reaction essential to carry flux towards the biomass equation is encoded by a single gene in the cancer-specific metabolic network because all other isoenzymes are not expressed in the cancer (loss of gene redundancy). This was the case of *AGPAT6*, and it was found by constraining the flux of the encoded reaction to zero in the generic human metabolic network from which the ccRCC metabolic network topology is derived. This constraint results in an unfeasible solution using the generic network, suggesting that at least one of the isoenzymes must be expressed to sustain biomass formation. In the second scenario, there are two alternative routes to support a flux towards the biomass equation, each encoded by a single gene, but just one is expressed in the cancer-specific metabolic network (loss of pathway redundancy). This was the case of *GALT*. To verify this, the reaction encoded by *GALT* was constrained to zero in the generic human metabolic network, and then a second round of single gene-knockouts was performed using the *GALT*-KO generic human metabolic network. In the end, the double *GALT*-*UGP2* knockout results in an unfeasible solution in the generic human metabolic network, indicative that *UGP2* encodes for a potential alternative pathway to *GALT* that is not expressed in ccRCC.
2. In the case of genes essential when using also the exchange fluxes to perform FBA, but non essential when the sole topology was used as a constraint, an unfeasible solution is trivially attributable to the implementation of one (or more) of these additional constraints. Therefore, for each essential gene, all constraints in (6) are released (set the lower and upper bound to –1000 and +1000 respectively) one at the time, until the metabolite whose constraint on the exchange flux caused unfeasibility is spotted. For *GSS* and *GCLC,* this metabolite is glutamate, while for *RRM2B* 3-ureidopropionate. Further interpretation of the individual mechanisms was achieved by following the fluxes around each key metabolite in the simulation, either when no knockout was applied or when the knockout was applied neglecting the constraint in the key metabolite exchange flux.

The fraction of ccRCC samples where a protein involved in any of the mechanisms above is stained with at least a weak signal was retrieved from the Human Protein Atlas v. 11^56^.

## Additional Information

**How to cite this article**: Gatto, F. *et al.* Flux balance analysis predicts essential genes in clear cell renal cell carcinoma metabolism. *Sci. Rep.* **5**, 10738; doi: 10.1038/srep10738 (2015).

## Supplementary Material

Supplementary Information

Supplementary Dataset 1

Supplementary Dataset 2

Supplementary Dataset 3

## Figures and Tables

**Table 1:**
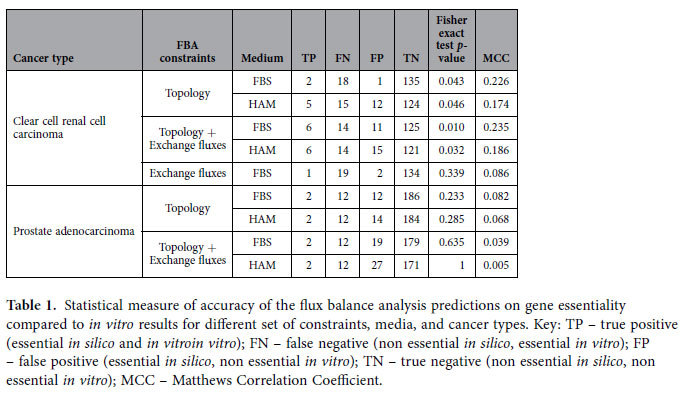
Statistical measure of accuracy of the flux balance analysis predictions on gene essentiality compared to *in vitro* results for different set of constraints, media, and cancer types
