# Supplementary material for: Flux balance analysis predicts essential genes in clear cell renal cell carcinoma metabolism
Source: Sci Rep. 2015 Jun 4;5:10738. doi: 10.1038/srep10738 (PMC4603759; doi:10.1038/srep10738)

Supplementary Information

Flux balance analysis predicts essential genes in clear cell renal cell carcinoma metabolism

Francesco Gatto[#], Heike Miess[#], Almut Schulze, Jens Nielsen.

Supplementary Figures

Supplementary Figure 1. The number of genes declared essential (red line) or non essential (blue line) *in vitro* for ccRCC according to the selected threshold for cell death in ≥ 70% of the cell lines (in terms of mean cell number reduction relative to control). The chosen threshold in this study is shown by the dotted line at 30%.

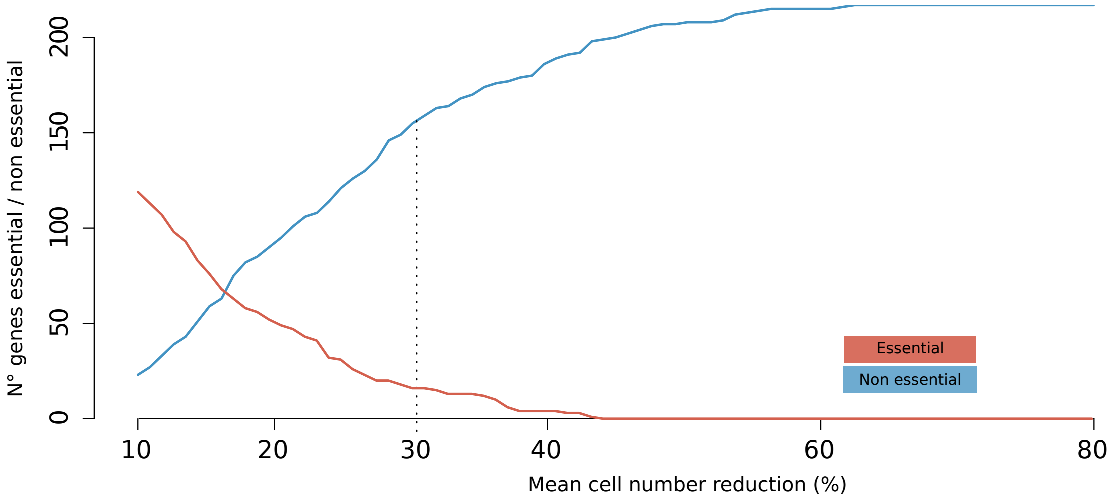

Supplementary Figure 2. Essential genes in ccRCC metabolism according to the functional RNAi screen. A gene is deemed essential (red) in ccRCC if the corresponding siRNA causes a ≥ 30% mean cell number reduction in ≥ 70% (4 out of 5) ccRCC cell lines compared to scrambled control. Triplicate wells per condition and cell line were performed.

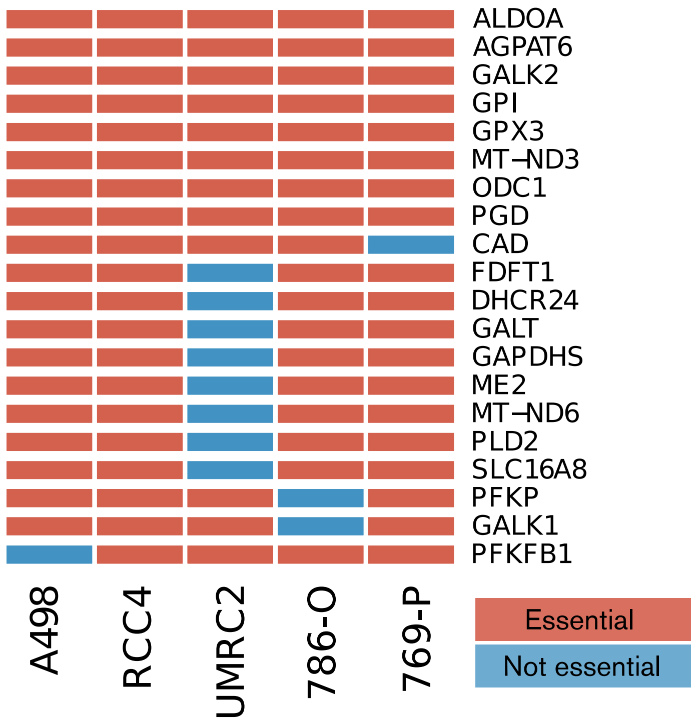

Supplementary Figure 3. Gene essentiality in ccRCC metabolism as predicted by flux balance analysis using the profile of exchange fluxes from seven ccRCC cell lines on top of a generic human network topology shows no accuracy in the reproduction of *in vitro* gene essentiality. A) Gene essentiality in ccRCC according to flux balance analysis using the profile of exchange fluxes from seven ccRCC cell lines on top of a generic human network topology. Only genes that are essential (red) using at least one flux profile are shown. Genes that are essential using at least 70% of the cell line flux profiles are deemed essential in ccRCC. B) Contingency table for the comparison between gene essentiality *in silico* vs. *in vitro* for those siRNAs in the library that had consensus effects in terms of cell number reduction in ≥ 70% of the cell lines. Only 1 true positive was found, *CAD*.

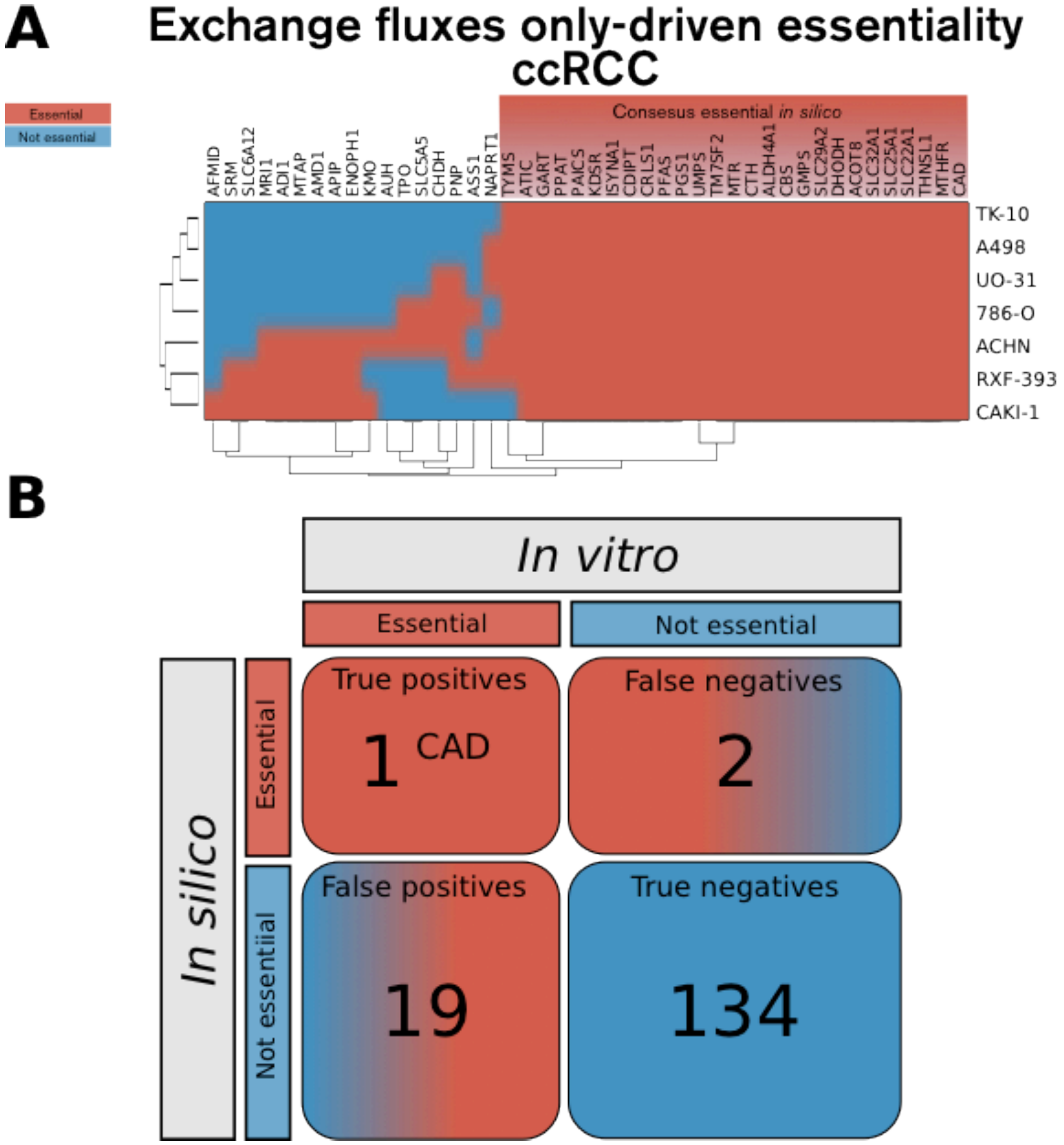

Supplementary Figure 4. Essential genes in PC metabolism according to a functional RNAi screen targeting ~230 metabolic enzymes, regulators and nutrient transporters. A gene is deemed essential (red) in PC if the corresponding siRNA causes a caspase activity *z*-score ≥ 2.5 in at least 2 out of 3 PC cell lines.

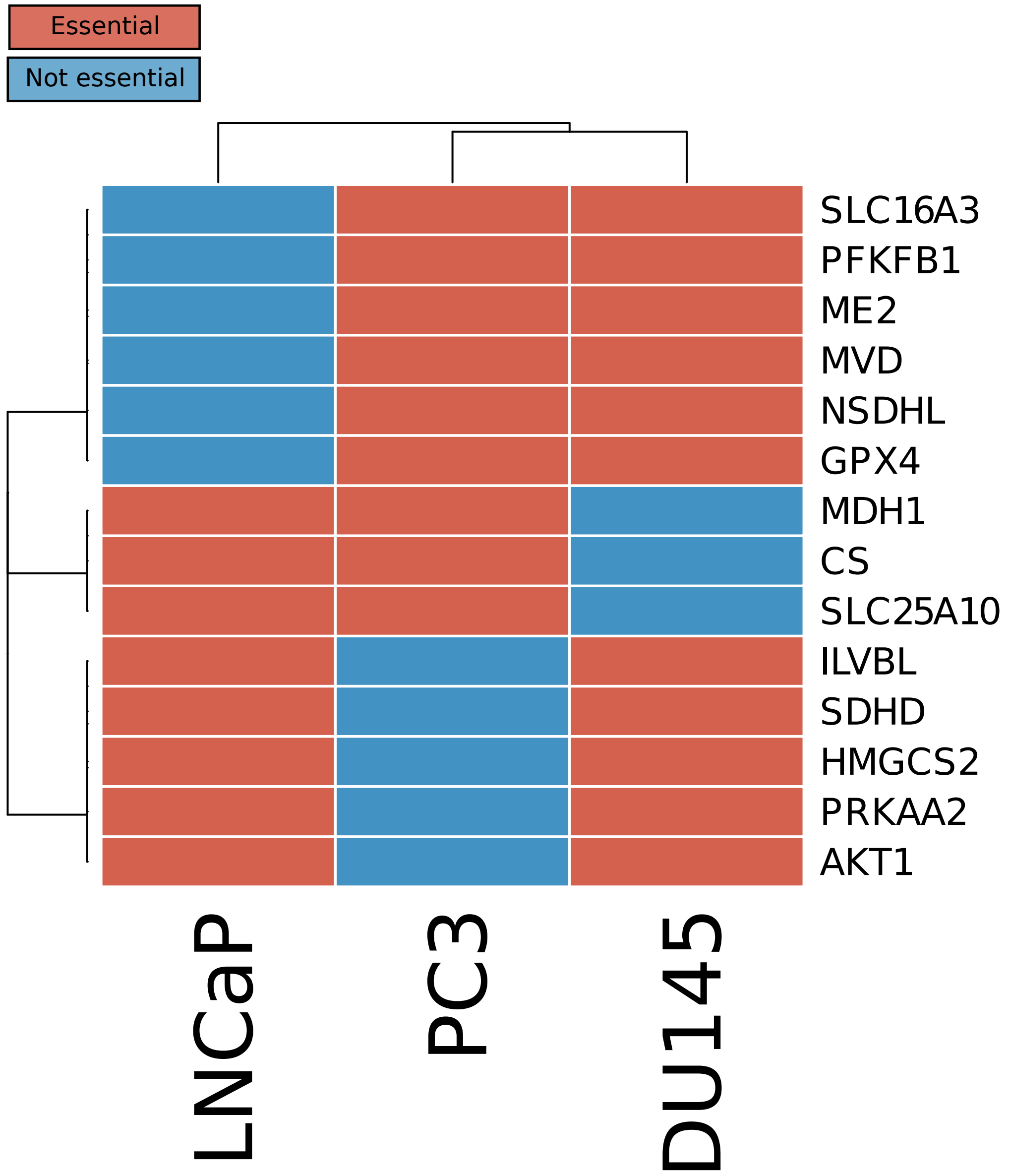

Supplementary Figure 5. Gene essentiality in PC metabolism as predicted by flux balance analysis using the metabolic network topology as a sole constraint for biomass formation (A) or by also implementing the profile of exchange fluxes from two PC cell lines (B). In the latter case, only genes that are essential using at least one flux profile are shown. Genes that are essential using both cell line flux profiles are deemed essential (red) in PC.

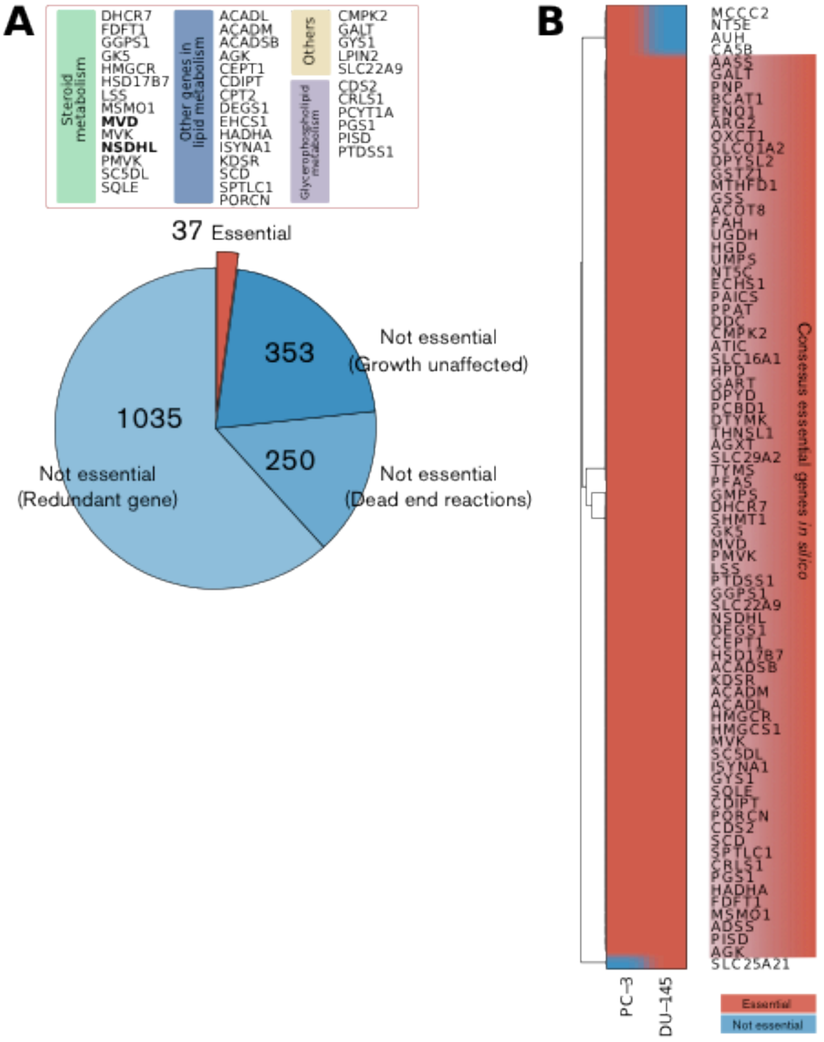

Supplementary Figure 6. Gene essentiality in PC metabolism as predicted by flux balance analysis using either the metabolic network topology alone or by implementing also the profile of exchange fluxes from two PC cell lines shows little accuracy in the comparison with the RNAi screen. A) Contingency table for the comparison between the declaration of gene essentiality *in silico* (using the metabolic network topology as the only constraint) vs. *in vitro* for those siRNAs in the library that had consensus effect in terms of caspase activity in ≥ 70% of the cell lines. MVD and NSDHL are true positives (p = 0.23). B) Boxplots of total caspase activity (as z-scores) for the groups of siRNAs predicted to be either essential (red) or non-essential (blue) *in silico* (using the metabolic network topology as the only constraint). C) Contingency table for the comparison between the declaration of gene essentiality *in silico* (using also the cell line flux profiles as constraints) vs. *in vitro* for those siRNAs in the library that had consensus effect in terms of caspa*GCLC, GSS, RRM2B* se activity in ≥ 70% of the cell lines. No novel true positives were detected. D) Boxplots of total caspase activity (as z-scores) for the groups of siRNAs predicted to be either essential (red) or non-essential (blue) *in silico* (using also the cell line flux profiles as constraints).

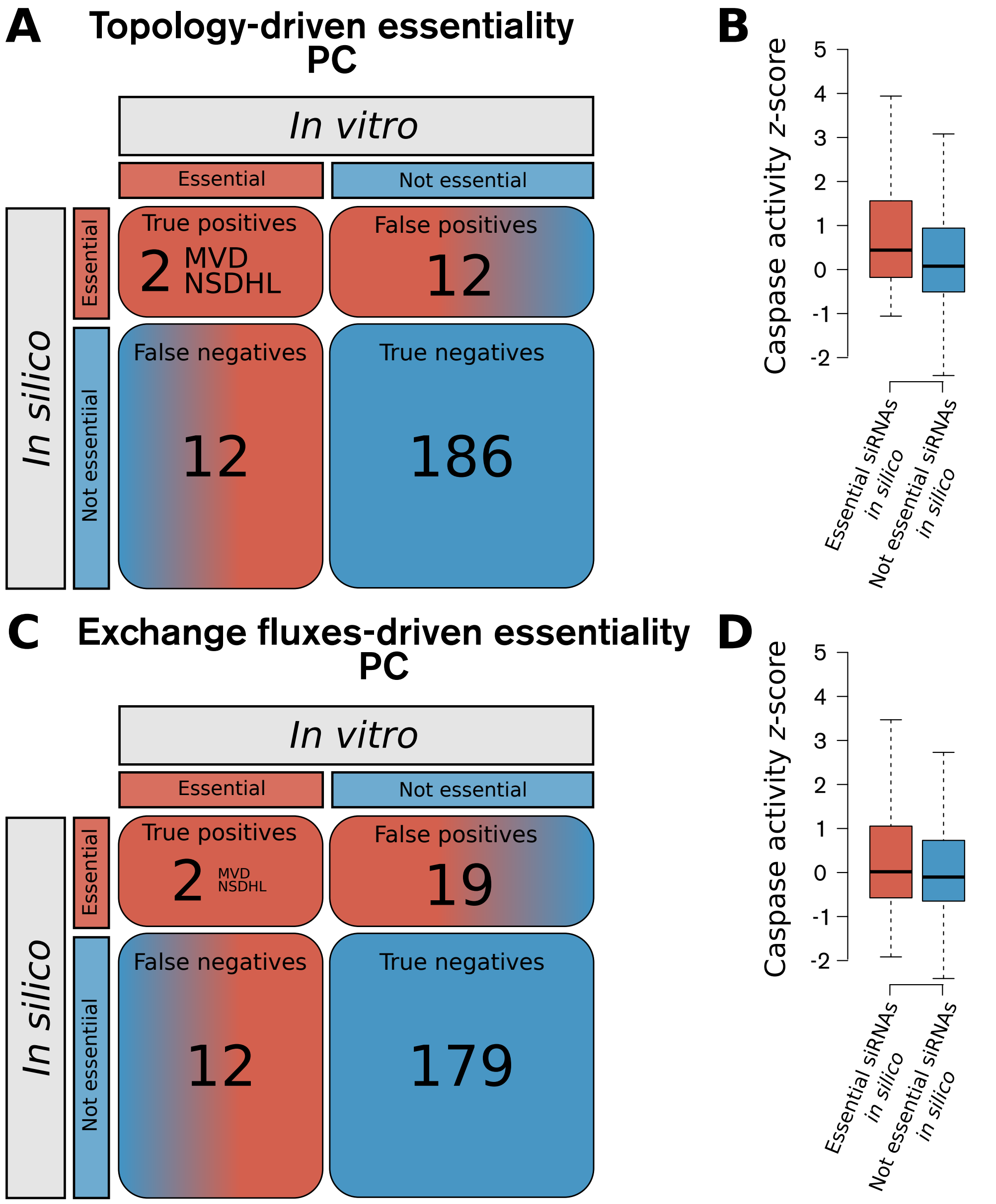

Supplementary Figure 7. Changes on gene essentiality in ccRCC according to flux balance analysis with a less permissive medium (HAM) and using the metabolic network topology as a sole constraint for biomass formation (A) or by also implementing the profile of exchange fluxes from seven ccRCC cell lines (B). In the latter case, only genes that are essential using at least one flux profile are shown. Genes that are essential using at least 70% of the cell line flux profiles are deemed essential in ccRCC.

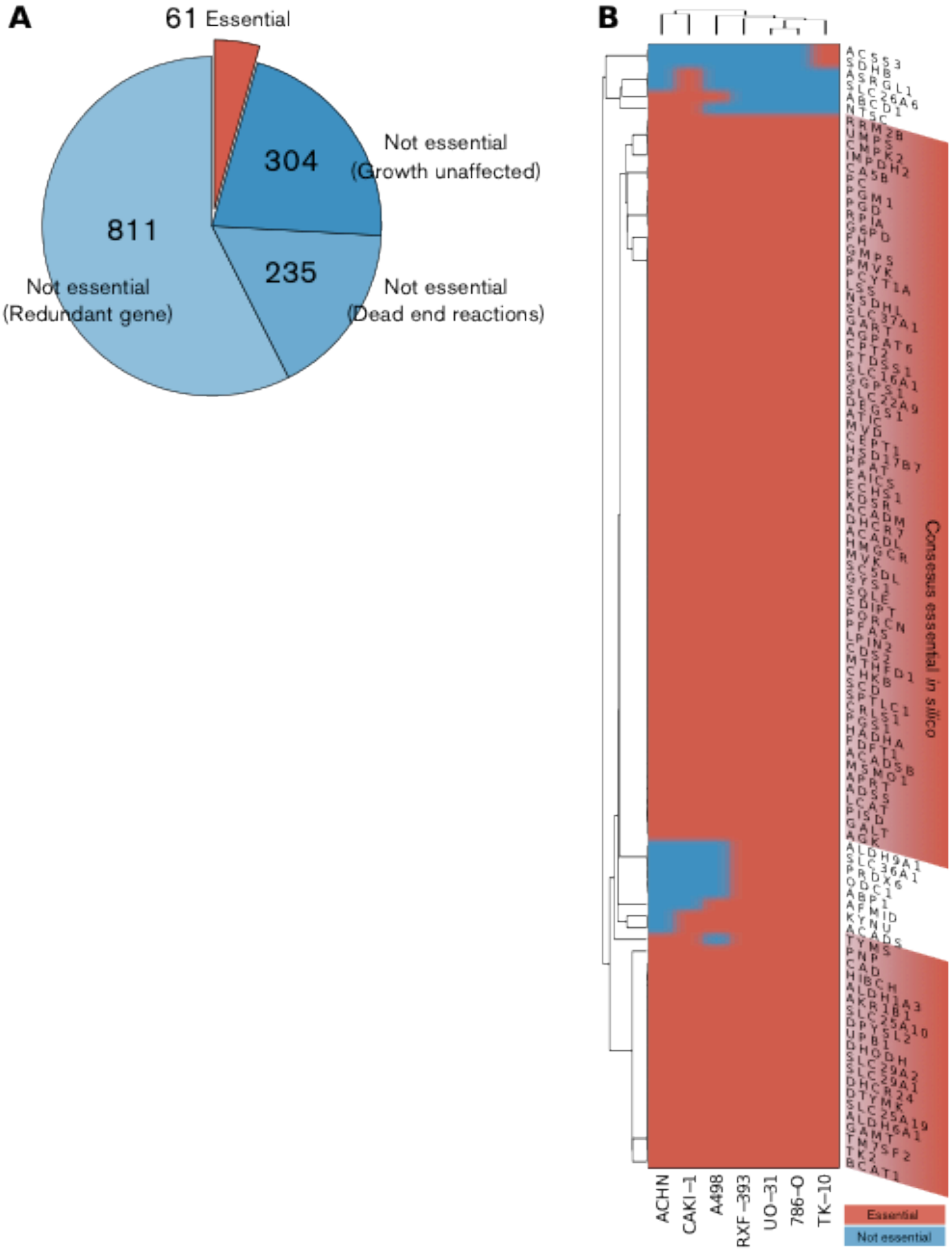

Supplementary Figure 8. Changes on gene essentiality in PC according to flux balance analysis with a less permissive medium (HAM) and using the metabolic network topology as a sole constraint for biomass formation (A) or by also implementing the profile of exchange fluxes from two PC cell lines (B). In the latter case, only genes that are essential using at least one flux profile are shown. Genes that are essential using both cell line flux profiles are deemed essential in PC.

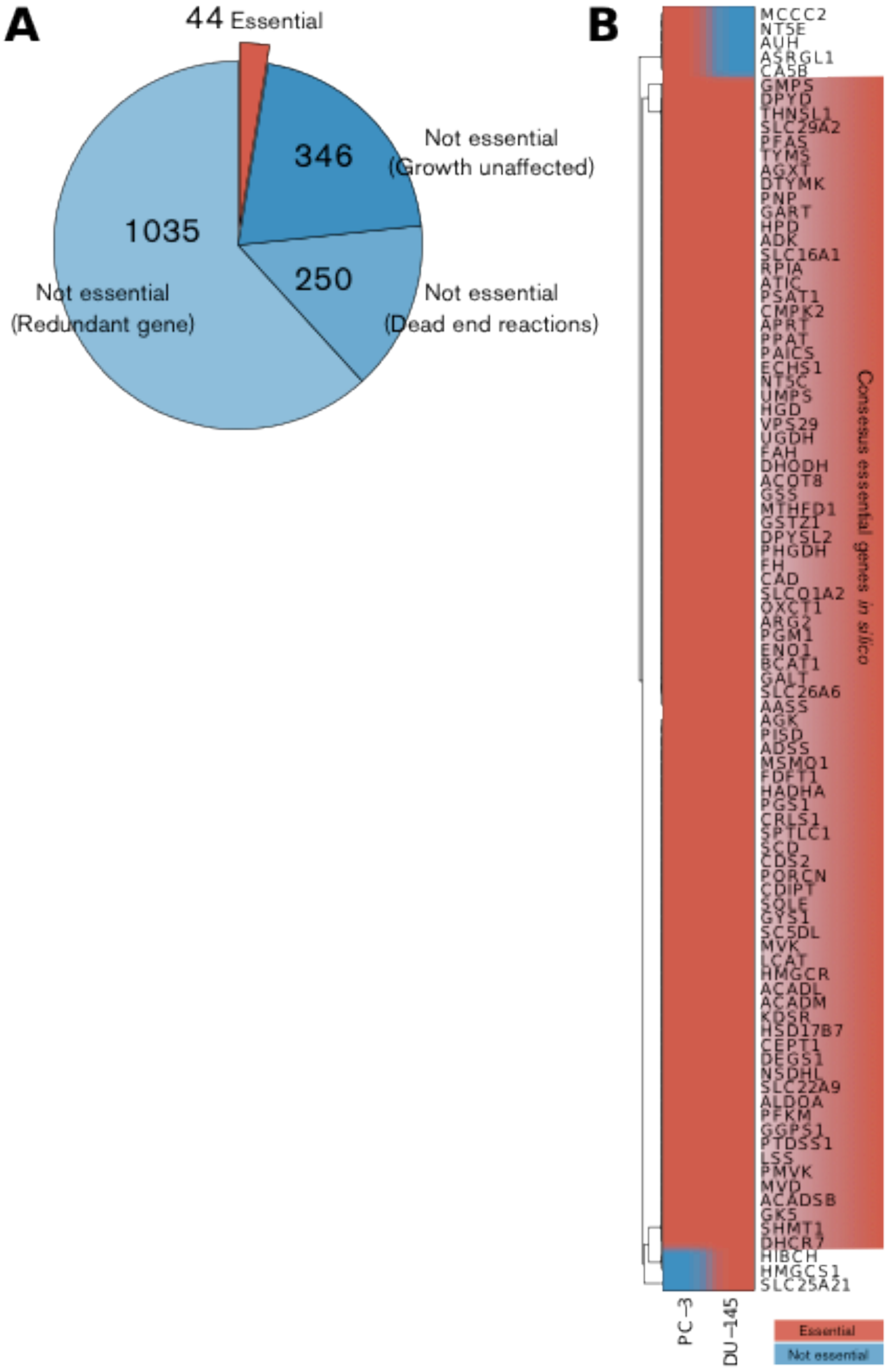

Supplementary Figure 9. Changes in the accuracy in the prediction of gene essentiality in cancer metabolism when choosing a less permissive medium composition (HAM) in flux balance analysis. A) Contingency table for the comparison between the declaration of ccRCC gene essentiality *in silico* vs. *in vitro* for those siRNAs in the library that had consensus effect in terms of cell number reduction in ≥ 70% of ccRCC cell lines. B) Boxplots of total cell number reduction for the groups of siRNAs predicted to be either essential or non-essential *in silico* for ccRCC. C) Contingency table for the comparison between the declaration of PC gene essentiality *in silico* vs. *in vitro* for those siRNAs in the library that had consensus effect in terms of caspase activity in 2 out of 3 PC cell lines. D) Boxplots of total caspase activity (as *z*-scores) for the groups of siRNAs predicted to be either essential or non essential *in silico* for PC.

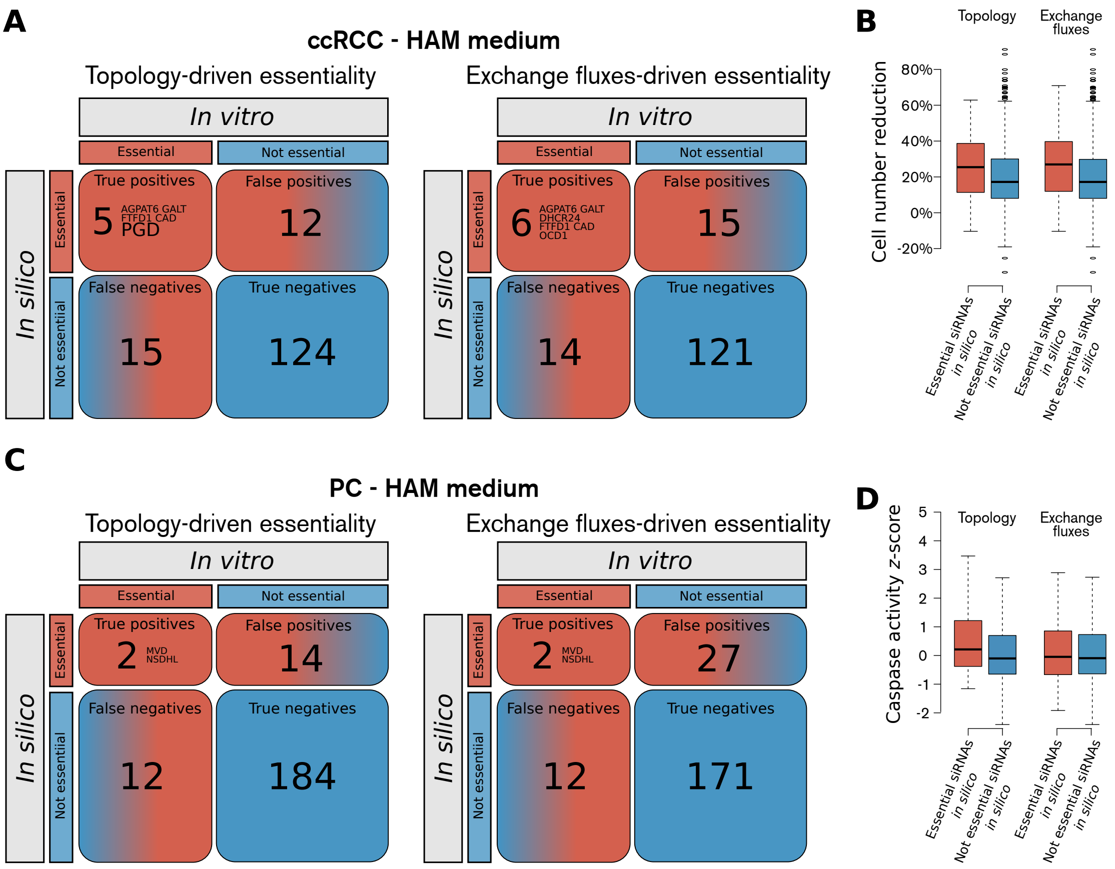

Supplementary Figure 10. Sensitivity analysis for the accuracy of the comparison between *in silico* essential genes in ccRCC and the RNAi screen at different thresholds of mean cell number reduction. The accuracy has been measured in terms of Matthews Correlation Coefficient, where a coefficient equal to 0 indicates a random classification between essential and non-essential genes by flux balance analysis. Also displayed is the number of *in vitro* essential genes with varying thresholds for the mean cell number reduction. The dotted line corresponds to the 30% threshold. A) Sensitivity analysis for the genes predicted by flux balance analysis with either rich (FBS, left) or less permissive (HAM, right) media and using the metabolic network topology alone as a constraint. B) Sensitivity analysis for the genes predicted by flux balance analysis with either rich (FBS, left) or less permissive (HAM, right) media using the profile of exchange fluxes from seven ccRCC cell lines in addition to ccRCC metabolic network topology as constraints.

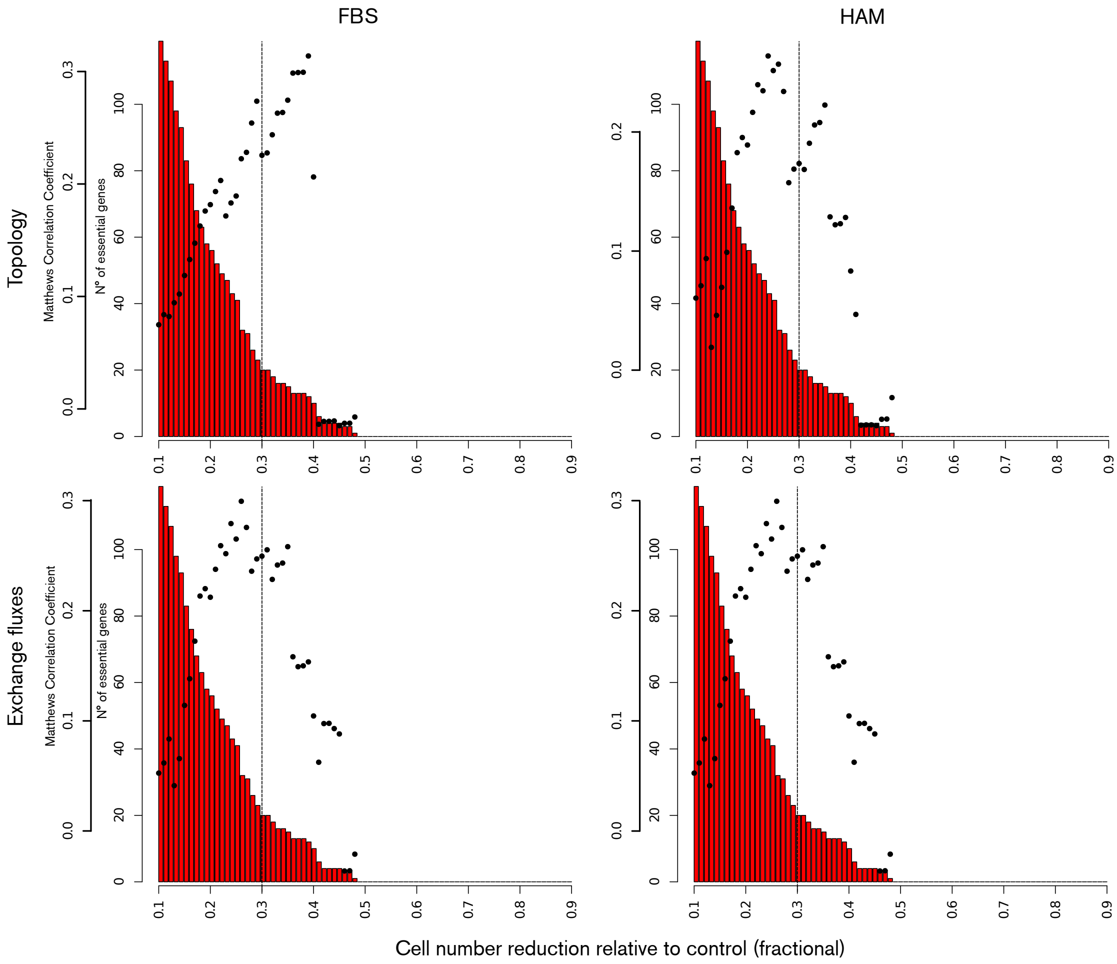

Supplementary Figure 11. Sensitivity analysis for the accuracy in the comparison between in silico essential genes in PC and the siRNA library at different threshold of caspase activity z-score (in the main text, a gene is deemed essential in PC if the corresponding siRNA causes a mean caspase activity z ≥ 2.5 in ≥ 70%, i.e. 2 out of 3 PC cell lines). The accuracy has been measured in terms of Matthews Correlation Coefficient, where a coefficient equal to 0 indicates a random classification between essential and non essential genes by flux balance analysis. Also displayed is the number of in vitro essential genes with varying threshold for the mean cell number reduction. The dotted line corresponds to the z = 2.5 threshold adopted in the main text. Top: sensitivity analysis for the genes predicted by flux balance analysis with either rich (FBS, left) or less permissive (HAM, right) media and using the metabolic network topology alone as a constraint. Bottom: sensitivity analysis for the genes predicted by flux balance analysis with either rich (FBS, left) or less permissive (HAM, right) media using the profile of exchange fluxes from two PC cell lines on top of PC metabolic network topology as constraints.

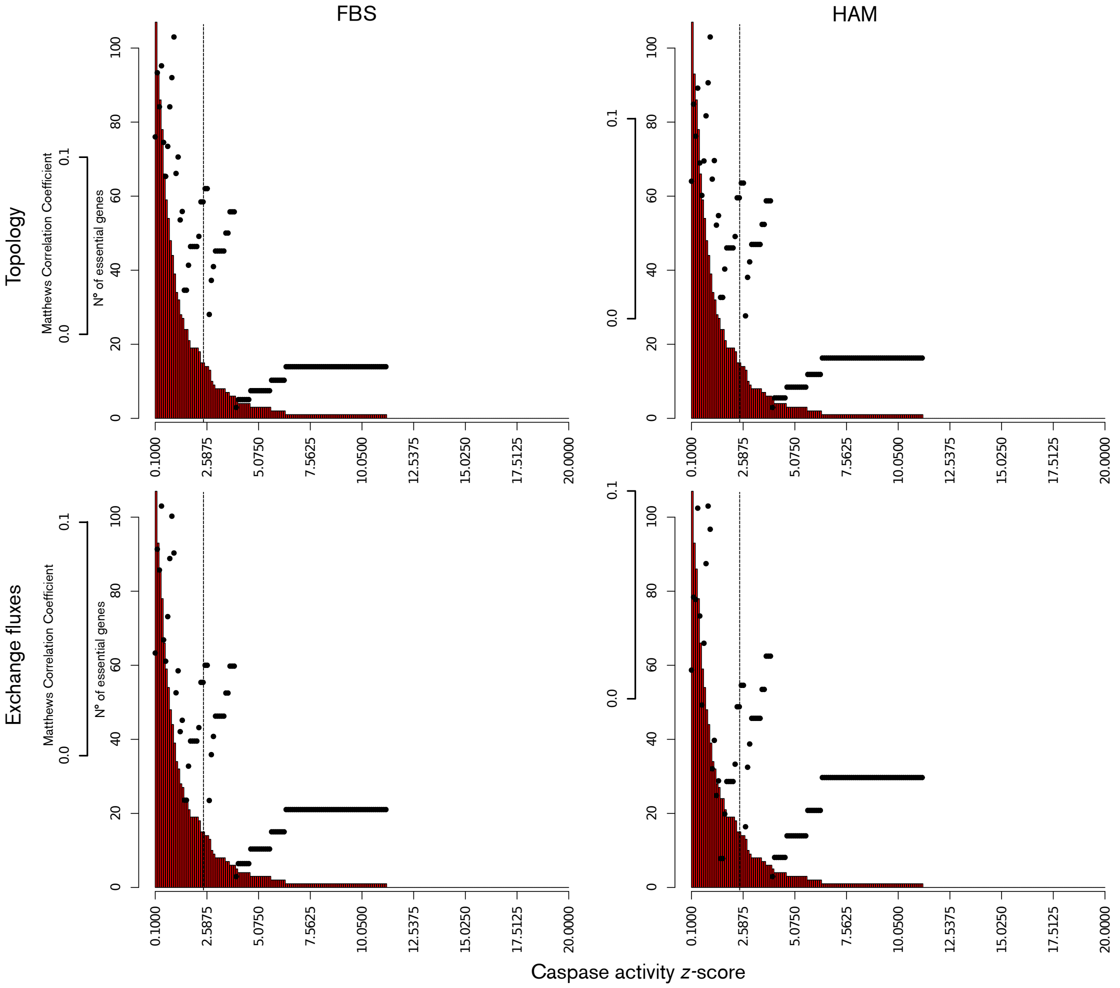

Supplementary Figure 12. Lower bounds on the uptake fluxes implemented in flux balance analysis for each profile of exchange fluxes when applied in rich (FBS) medium using the ccRCC metabolic network topology. Differences in flux profiles are reflected by differences in the bounds that can therefore explain a differential susceptibility in single gene knockouts. Fluxes are in fmol cell^{-1} h^{-1} and they were retrieved from [50] and adjusted as suggested in [49]. Blue scales with the magnitude of secretion fluxes, while red of uptake fluxes.

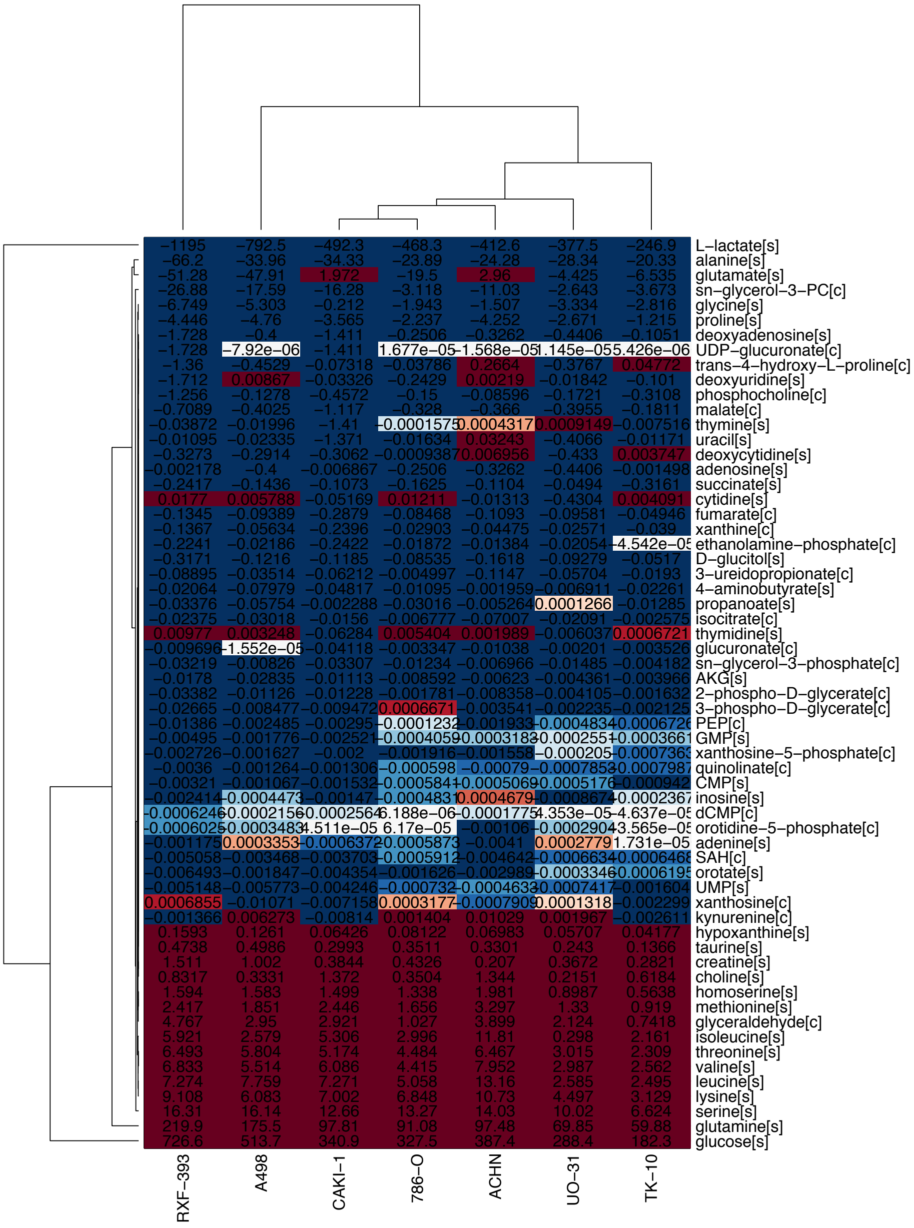

Supplementary Figure 13. Upper bounds on the uptake fluxes implemented in flux balance analysis for each profile of exchange fluxes when applied in rich (FBS) medium using the ccRCC metabolic network topology. Differences in flux profiles are reflected by differences in the bounds that can therefore explain a differential susceptibility in single gene knockouts. Fluxes are in fmol cell^{-1} h^{-1} and they were retrieved from [50] and adjusted as suggested in [49].

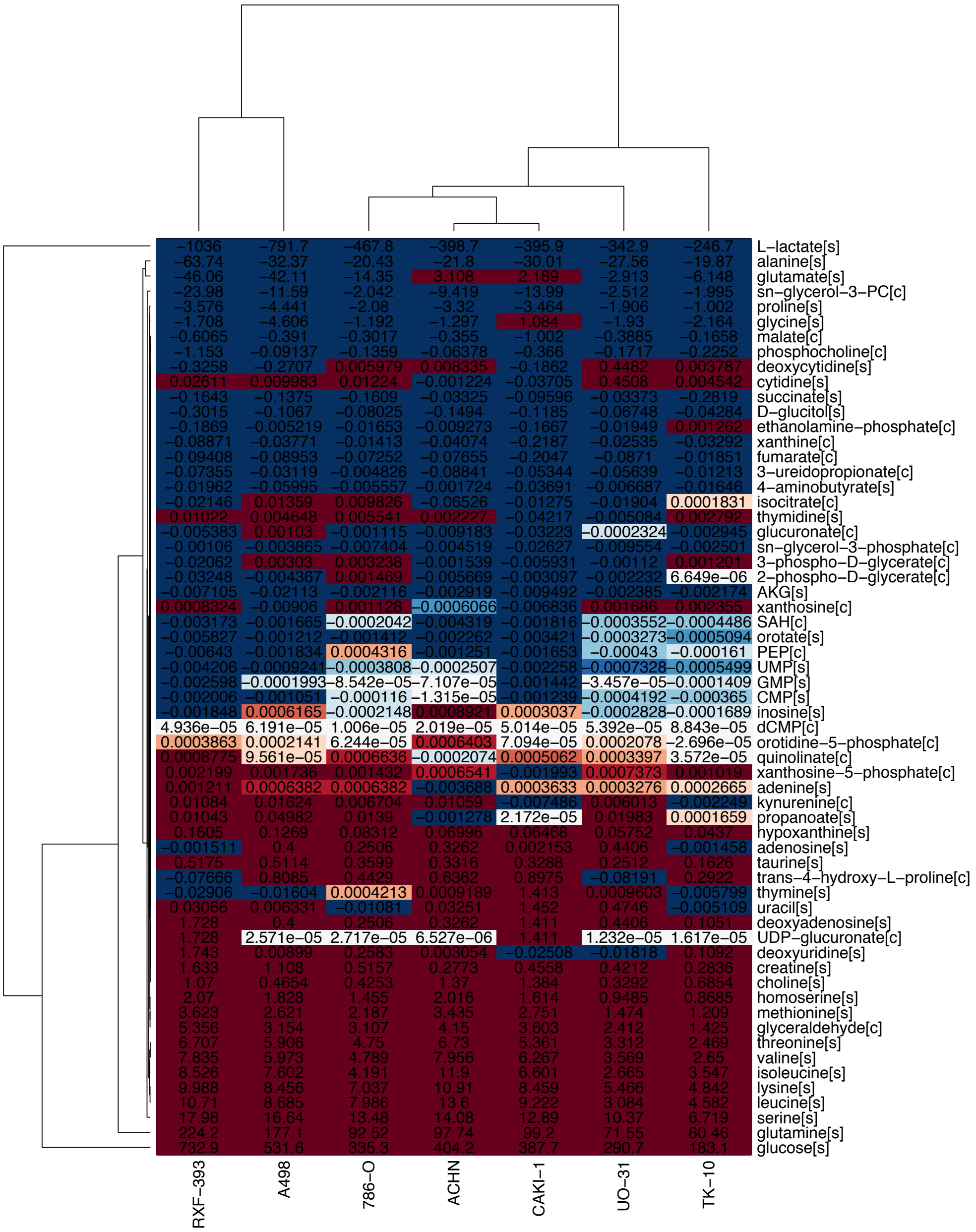

Supplementary Figure 14. Lower bounds on the uptake fluxes implemented in flux balance analysis for each profile of exchange fluxes when applied in HAM using the ccRCC metabolic network topology. Differences in flux profiles are reflected by differences in the bounds that can therefore explain a differential susceptibility in single gene knockouts. Fluxes are in fmol cell^{-1} h^{-1} and they were retrieved from [50] and adjusted as suggested in [49]. Blue scales with the magnitude of secretion fluxes, while red of uptake fluxes.

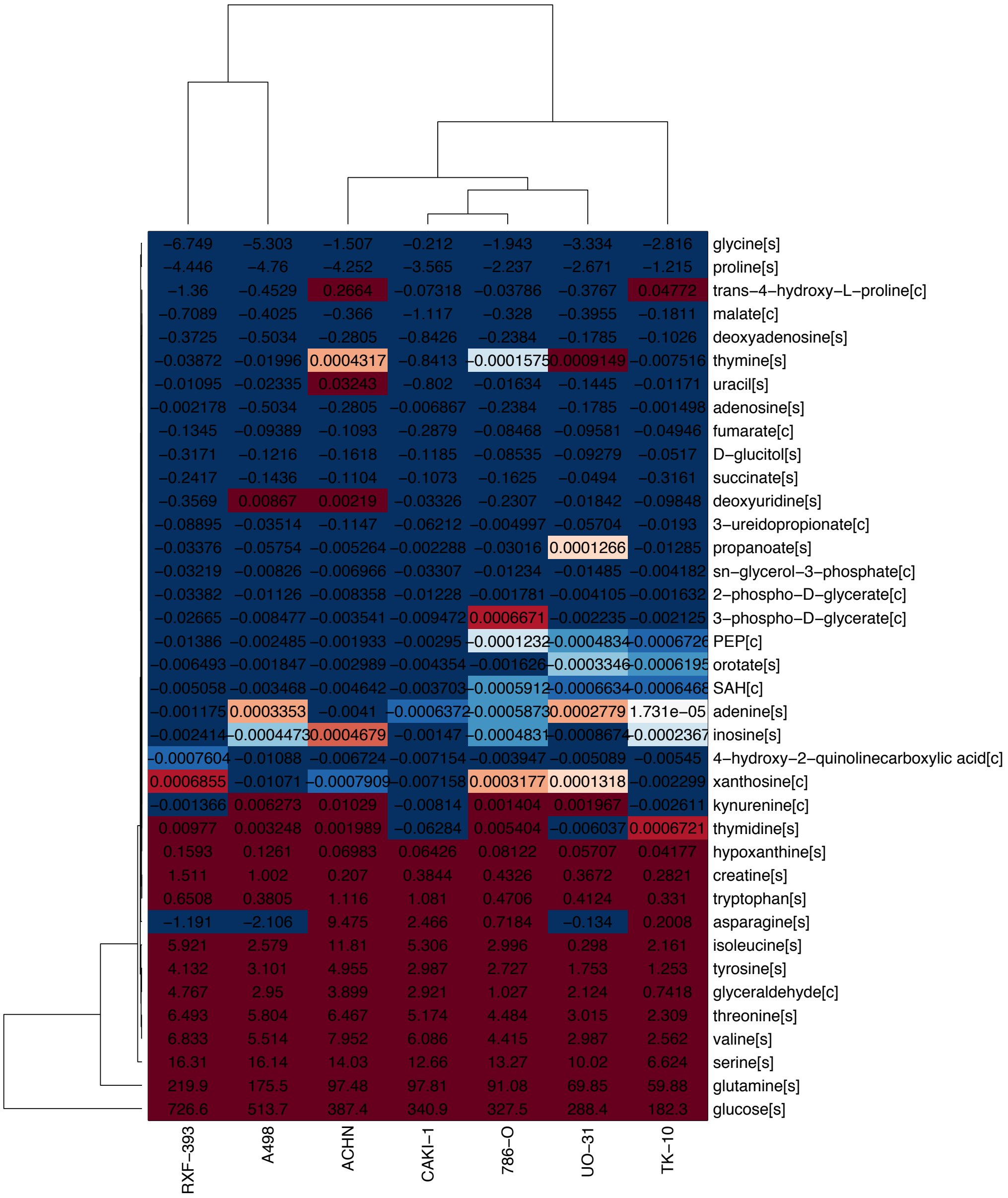

Supplementary Figure 15. Upper bounds on the uptake fluxes implemented in flux balance analysis for each profile of exchange fluxes when applied in HAM using the ccRCC metabolic network topology. Differences in flux profiles are reflected by differences in the bounds that can therefore explain a differential susceptibility in single gene knockouts. Fluxes are in fmol $cell^{-1}$ h^{-1} and they were retrieved from [50] and adjusted as suggested in [49]. Blue scales with the magnitude of secretion fluxes, while red of uptake fluxes.

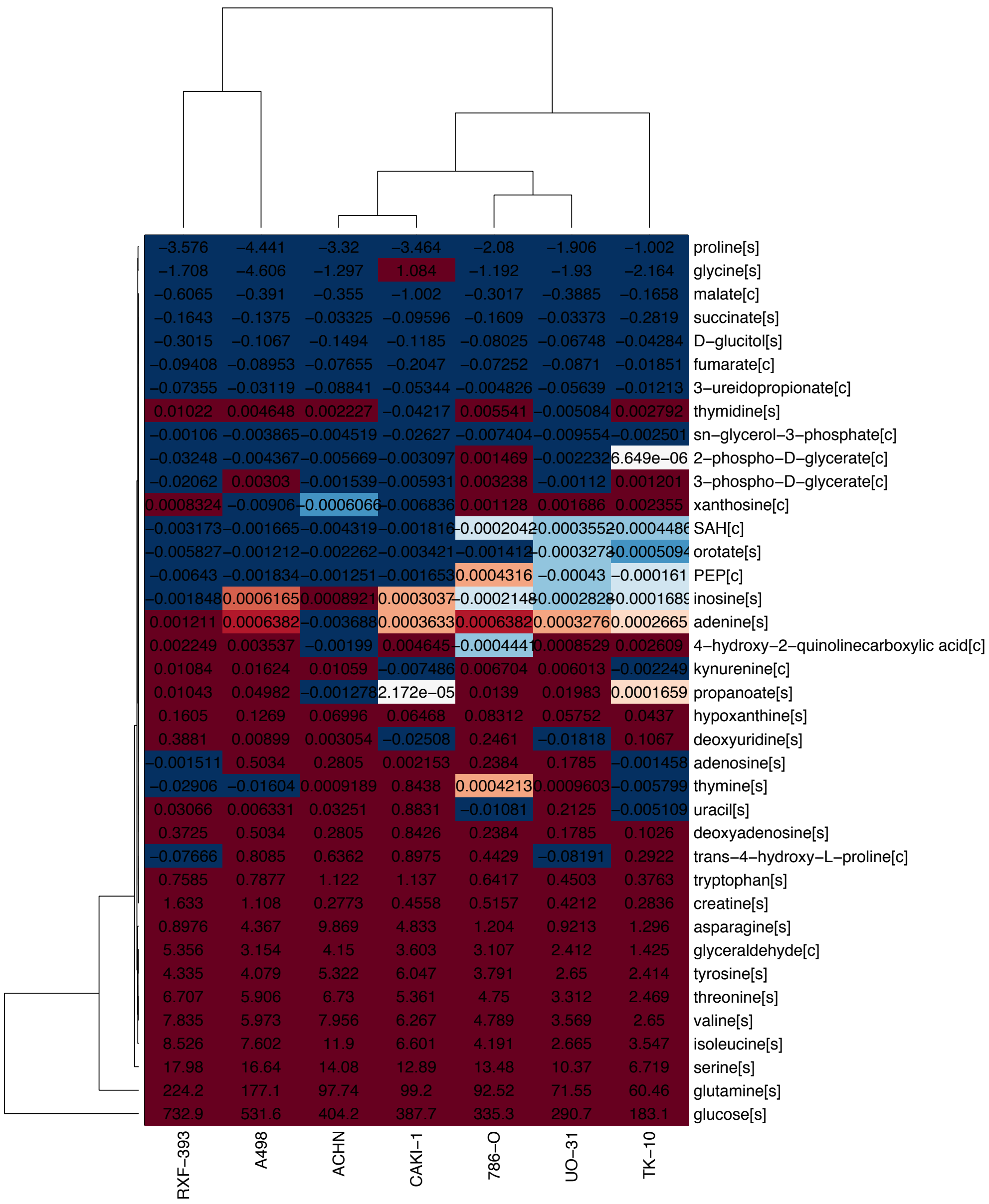

Supplementary Figure 16. Lower bounds on the uptake fluxes implemented in flux balance analysis for each profile of exchange fluxes when applied in rich (FBS) medium using the PC metabolic network topology. Differences in flux profiles are reflected by differences in the bounds that can therefore explain a differential susceptibility in single gene knockouts. Fluxes are in fmol cell^{-1} h^{-1} and they were retrieved from [50] and adjusted as suggested in [49]. Blue scales with the magnitude of secretion fluxes, while red of uptake fluxes.

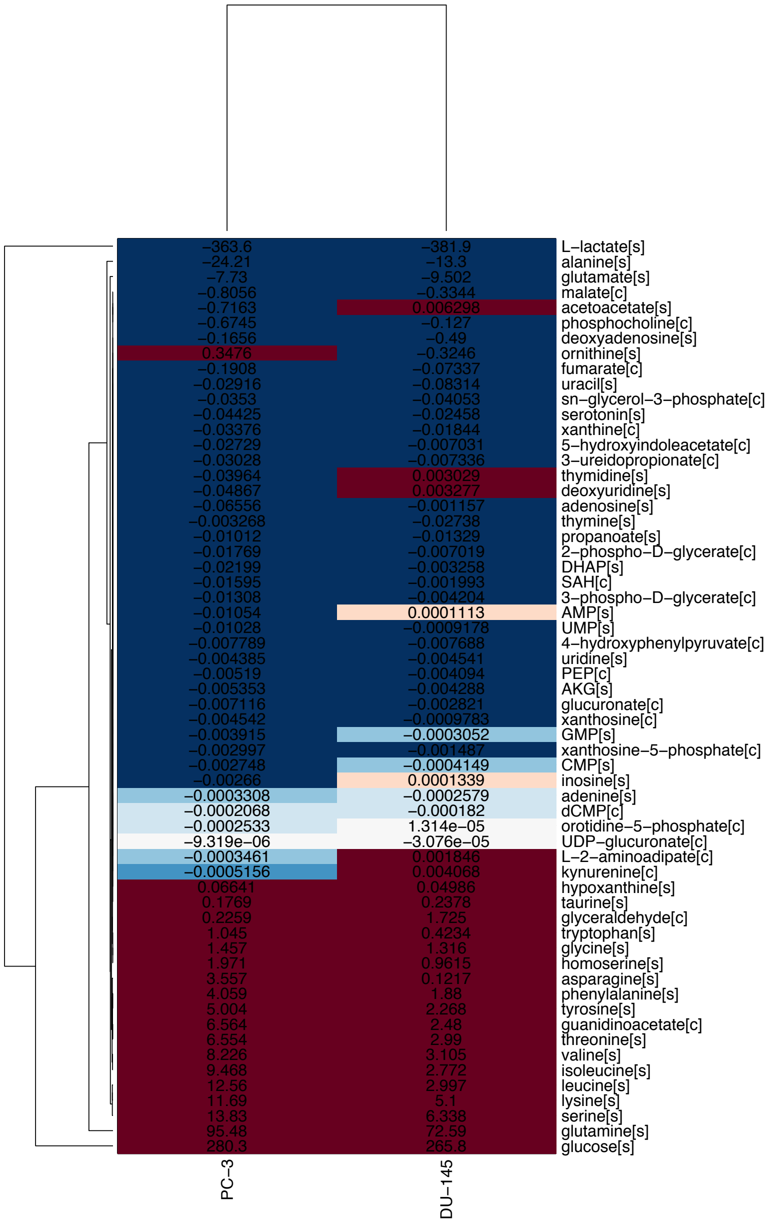

Supplementary Figure 17. Upper bounds on the uptake fluxes implemented in flux balance analysis for each profile of exchange fluxes when applied in rich (FBS) medium using the PC metabolic network topology. Differences in flux profiles are reflected by differences in the bounds that can therefore explain a differential susceptibility in single gene knockouts. Fluxes are in fmol cell^{-1} h^{-1} and they were retrieved from [50] and adjusted as suggested in [49]. Blue scales with the magnitude of secretion fluxes, while red of uptake fluxes.

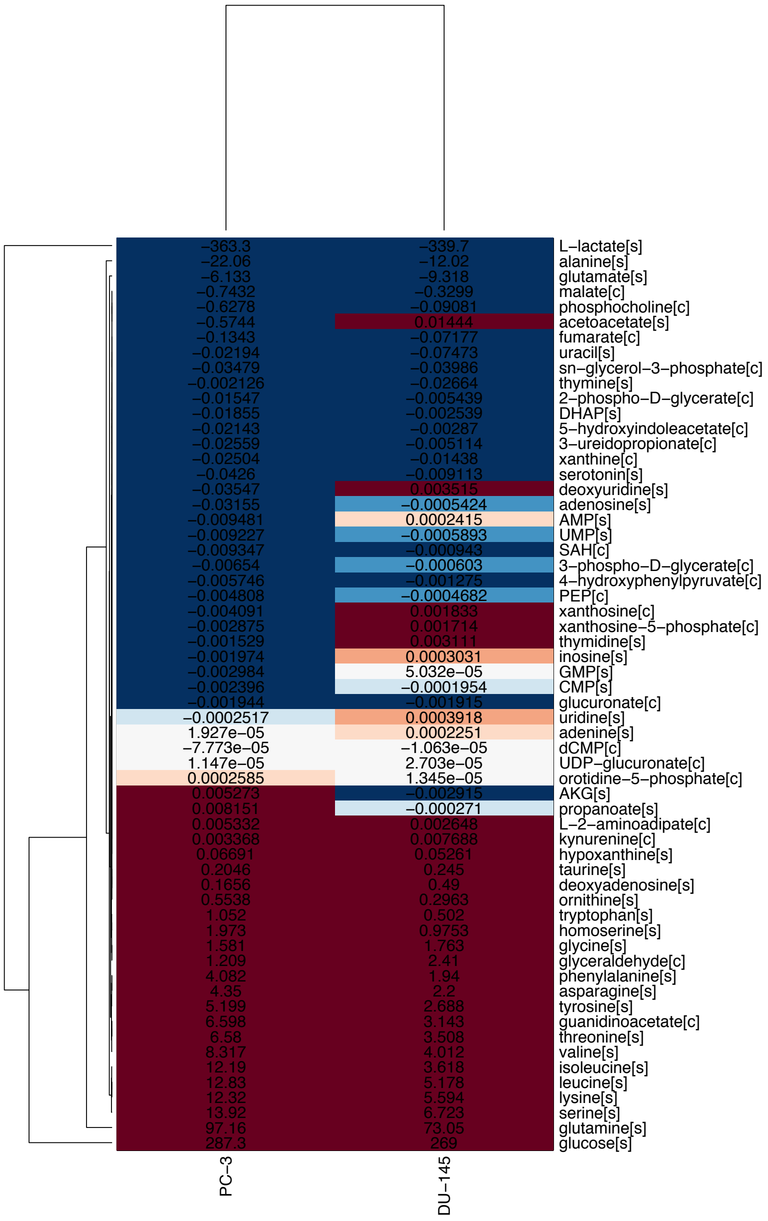

Supplementary Figure 18. Lower bounds on the uptake fluxes implemented in flux balance analysis for each profile of exchange fluxes when applied in HAM using the PC metabolic network topology. Differences in flux profiles are reflected by differences in the bounds that can therefore explain a differential susceptibility in single gene knockouts. Fluxes are in fmol cell^{-1} h^{-1} and they were retrieved from [50] and adjusted as suggested in [49]. Blue scales with the magnitude of secretion fluxes, while red of uptake fluxes.

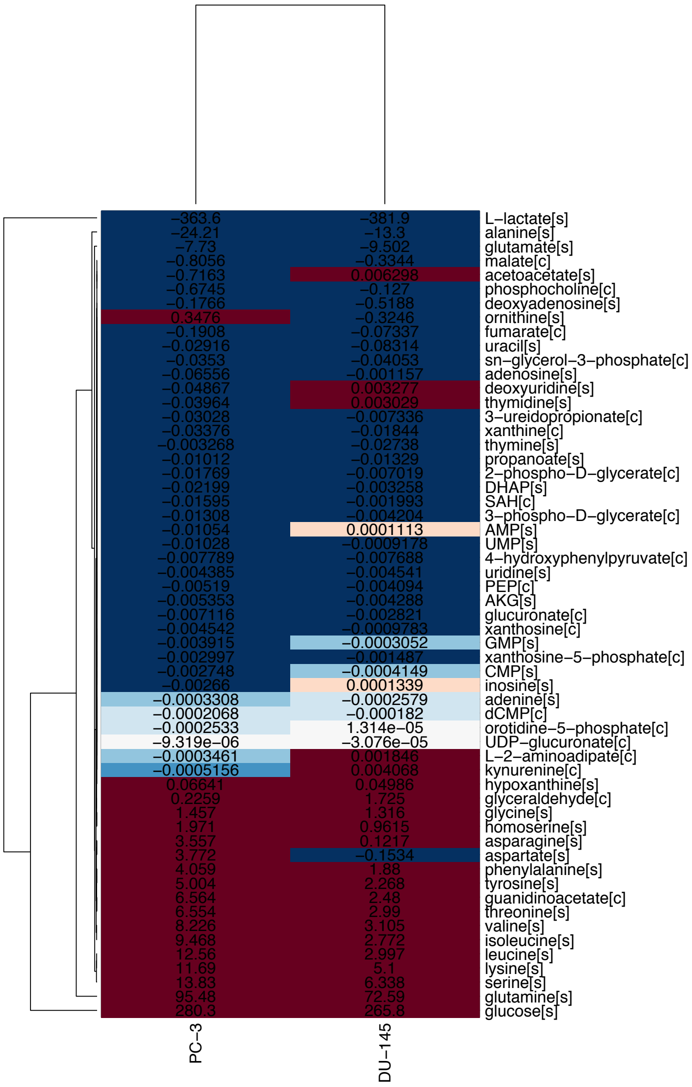

Supplementary Figure 19. Upper bounds on the uptake fluxes implemented in flux balance analysis for each profile of exchange fluxes when applied in HAM using the PC metabolic network topology. Differences in flux profiles are reflected by differences in the bounds that can therefore explain a differential susceptibility in single gene knockouts. Fluxes are in fmol cell^{-1} h^{-1} and they were retrieved from [50] and adjusted as suggested in [49]. Blue scales with the magnitude of secretion fluxes, while red of uptake fluxes.

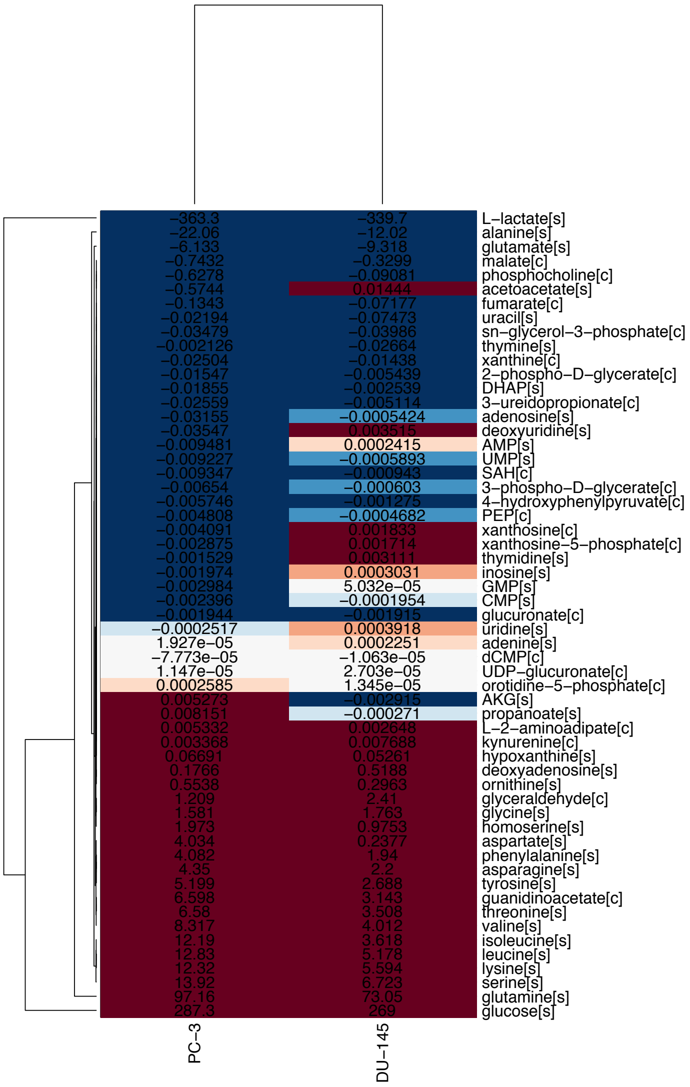

Supplement: Supplementary Information [file srep10738-s1.pdf]
